# Metabolomics Profiling of Stages of Coronary Artery Disease Progression

**DOI:** 10.3390/metabo14060292

**Published:** 2024-05-22

**Authors:** Gulsen Guliz Anlar, Najeha Anwardeen, Sarah Al Ashmar, Shona Pedersen, Mohamed A. Elrayess, Asad Zeidan

**Affiliations:** 1 Department of Basic Medical Sciences, College of Medicine, QU Health, Qatar University, Doha P.O. Box 2713, Qatar; ga1912359@student.qu.edu.qa (G.G.A.); sa1903822@student.qu.edu.qa (S.A.A.); spedersen@qu.edu.qa (S.P.); 2 Biomedical Research Center (BRC), QU Health, Qatar University, Doha P.O. Box 2713, Qatar; n.anwardeen@qu.edu.qa (N.A.); m.elrayess@qu.edu.qa (M.A.E.)

**Keywords:** metabolomics, coronary artery disease, hypercholesterolemia, dyslipidemia, diabetes, hypertension, blood pressure, biomarkers, atherosclerosis

## Abstract

Coronary artery disease (CAD) and atherosclerosis pose significant global health challenges, with intricate molecular changes influencing disease progression. Hypercholesterolemia (HC), hypertension (HT), and diabetes are key contributors to CAD development. Metabolomics, with its comprehensive analysis of metabolites, offers a unique perspective on cardiovascular diseases. This study leveraged metabolomics profiling to investigate the progression of CAD, focusing on the interplay of hypercholesterolemia, hypertension, and diabetes. We performed a metabolomic analysis on 221 participants from four different groups: (I) healthy individuals, (II) individuals with hypercholesterolemia (HC), (III) individuals with both HC and hypertension (HT) or diabetes, and (IV) patients with self-reported coronary artery disease (CAD). Utilizing data from the Qatar Biobank, we combined clinical information, metabolomic profiling, and statistical analyses to identify key metabolites associated with CAD risk. Our data identified distinct metabolite profiles across the study groups, indicating changes in carbohydrate and lipid metabolism linked to CAD risk. Specifically, levels of mannitol/sorbitol, mannose, glucose, and ribitol increased, while pregnenediol sulfate, oleoylcarnitine, and quinolinate decreased with higher CAD risk. These findings suggest a significant role of sugar, steroid, and fatty acid metabolism in CAD progression and point to the need for further research on the correlation between quinolinate levels and CAD risk, potentially guiding targeted treatments for atherosclerosis. This study provides novel insights into the metabolomic changes associated with CAD progression, emphasizing the potential of metabolites as predictive biomarkers.

## 1. Introduction

Coronary artery disease (CAD) and atherosclerosis are complex multifactorial cardiovascular diseases with a significant global health impact. CAD results from the narrowing of the coronary arteries due to the accumulation of atherosclerotic plaques and is a leading cause of morbidity and mortality worldwide [1]. Atherosclerosis itself is marked by the gradual formation of lipid-laden plaques within the arterial walls, impeding blood flow and potentially resulting in heart attacks and strokes [2]. This disease emerges from a combination of genetic influences, environmental factors, and personal lifestyle choices, with hypercholesterolemia (HC), hypertension (HT), and diabetes being prominent factors in its development.

The progression from health to CAD involves intricate molecular changes, including disruptions in lipid metabolism, inflammation, oxidative stress, and endothelial dysfunction [3]. HC, characterized by elevated levels of lipids in the bloodstream, is a well-established risk factor for both CAD and atherosclerosis [4]. Accumulating evidence from epidemiological studies and clinical trials has consistently shown a clear link between high levels of low-density lipoprotein cholesterol (LDL-C) and an increased risk of developing CAD and atherosclerosis [5,6]. Moreover, genetic studies on the PCSK9 gene mutation have further clarified the direct connection between high LDL-C and the risk for CAD [7], leading to LDL-C reduction through drugs like statins as a key prevention strategy for CAD [8].

Diabetes, encompassing both type 1 and type 2, doubles the risk of CAD, mirroring the risk posed by a prior heart attack [9]. It triggers chronic high blood sugar and insulin resistance, leading to a cascade of damaging effects, including the production of advanced glycation end products and oxidative stress, fostering conditions that encourage atherosclerosis [10,11,12]. Similarly, elevated blood pressure (BP) also promotes endothelial dysfunction, inflammation, and oxidative stress, ultimately accelerating the atherosclerotic process and increasing the risk of plaque rupture [13]. Large-scale cohort studies such as the Framingham Heart Study have shown a robust correlation between hypertension and the occurrence of CAD [14]. The combined presence of HT and diabetes, especially when accompanied by hyperlipidemia, aggravates the cardiovascular risk profile [15].

It is important to acknowledge that not all at-risk individuals will develop atherosclerosis. Despite extensive research on CVD, there is still a pressing need to predict these diseases before they cause severe consequences. Recent studies have pinpointed several biomarkers, including hemoglobin A1c (HbA1c), uric acid, high-sensitivity C-reactive protein (hs-CRP), the triglyceride-glucose (TyG) index, and the atherogenic index of plasma (AIP), that can independently predict coronary lesions and the potential for future atherosclerosis events [16,17,18,19,20].

Metabolomics, with its holistic examination of metabolites in biological samples, offers a significant advantage over conventional single-biomarker studies. This approach has emerged as a valuable tool in unraveling the intricate molecular mechanisms underlying cardiovascular diseases and identifying potential biomarkers for risk stratification [21]. By detecting changes in metabolite levels, metabolomics can provide insights into metabolic changes associated with disease states, pointing to potential therapeutic targets and enhancing diagnostic methods.

In light of the above, our study seeks to perform an extensive analysis of metabolomics data from four distinct populations: healthy individuals, those with hyperlipidemia, those with both hyperlipidemia and hypertension or diabetes, and CAD patients. Utilizing sophisticated analytical techniques, we aim to pinpoint specific metabolites that show notable concentration changes correlating with an increased risk of CAD and atherosclerosis. By uncovering novel metabolic processes and biomarkers associated with the progression from HC, HT, and diabetes to CAD, this research has the potential to enhance our comprehension of the pathophysiology of CAD and atherosclerosis. Ultimately, this understanding could pave the way for the creation of personalized medical approaches and targeted therapies to mitigate the effects of CVDs; however, additional research is required to translate these results into clinical practice.

## 2. Materials and Methods

### 2.1. The Study Population

Clinical information and metabolomic data were provided by the Qatar Biobank (QBB). The metabolomics data set used in this study can be found in Supplementary Table S1. This study comprised 40 healthy controls with no chronic disease, 32 CAD patients, 35 HC patients, and 114 HC + complications patients with available metabolomics data. The inclusion criteria for the control group included healthy adults; for the HC group, they included adults with LDL cholesterol levels above 3.36 mmol/L (130 mg/dL); for the HC + complications group, they included adults with HC and systolic BP > 139 mmHg or diastolic BP > 89 mmHg or HbA1c > 6.4%, and for the CAD group, they included adults with a self-reported previous diagnosis of angina or heart attack. The exclusion criteria for the control and HC groups were blood HbA1c > 6.4%, systolic BP > 139 mmHg, or diastolic BP > 89 mmHg. All participants in line with the inclusion and exclusion criteria, with available metabolomics data provided by the Qatar Biobank, were included in this study. This investigation was approved by the Institutional Review Board of the Qatar Biobank, Doha, Qatar (IRB protocol no: E-2021-QF-QBB-RES-ACC-00022-0163).

### 2.2. Sample Collection and Processing

Detailed information regarding the collection of biological specimens was previously given [22]. Each participant had 60 milliliters of blood drawn. A portion of the blood was analyzed for clinical markers, while the rest was divided into smaller amounts and stored in microtubes. These samples were kept at −80 °C for short-term use and in the liquid nitrogen vapor phase for long-term storage. The blood samples were centrifuged to separate the white blood cells, red blood cells, and plasma containing EDTA.

### 2.3. Biochemical and Biometric Analysis

Participants’ blood samples were taken to determine the levels of biochemical biomarkers such as C-reactive protein, thyroid hormone, blood glucose, liver and kidney function, and total blood count. Hematology and blood biochemistry were analyzed in the laboratory at Doha’s Hamad Medical Center [22]. Using a Seca stadiometer, the height and weight of each participant were calculated. The VICORDER gadget was used to measure the pulse wave velocity parameter, which is used to evaluate arterial stiffness. Using the automated Omron 705 device, two diastolic and systolic blood pressure readings were recorded, and a third reading was taken if there was a difference of at least 5 mmHg. The average BP values were used in this study.

### 2.4. Metabolomics Profiling

Metabolic profiling was carried out in accordance with recognized procedures at Metabolon, Durham, NC, USA. In all cases, a heated electrospray ionization (HESI-II) source and an Orbitrap mass analyzer with a 35,000 mass resolution were used in conjunction with a Waters ACQUITY ultra-performance liquid chromatography (UPLC) system (Waters, Milford, MA, USA) and a Thermo Scientific Q-Exactive high-resolution/accurate mass spectrometer (Waltham, MA, USA). The liquid chromatography–mass spectrometry (LC-MS) methodology was previously explained in detail [23]. Briefly, the protein fraction was isolated from serum samples using methanol. The resulting extract was divided into five fractions: two for analysis using reverse phase (RP)/UPLC-MS/MS methods with positive ion mode electrospray ionization (ESI), one for analysis using RP/UPLC-MS/MS with negative ion mode ESI, one for analysis using hydrophilic interaction chromatography (HILIC)/UPLC-MS/MS with negative ion mode ESI, and one sample was set aside as a backup. Utilizing the hardware and software from Metabolon (Durham, NC, USA), raw data were retrieved, peaks were detected, and quality control was performed. With more than 3300 commercially available, purified standard chemicals, substances were identified by comparison to library entries of purified standards or recurrent unknown items. Each sample’s library matches for each compound were examined, and any errors were fixed as necessary.

### 2.5. Statistical Analysis

Demographic and clinical information and data were classified into four groups: control, HC, HC with complications, and CAD. Propensity score matching was performed using the R package “MatchIt” to match age and gender between the groups. The method “optimal” was employed to ensure that the sum of the absolute pairwise distance in the matched pairs was as small as possible. Optimally matched data were utilized for the subsequent downstream analysis. The measured variables were presented as the mean and standard deviation (SD) for parametric values; median with interquartile ratio (IQR) for non-parametric values; and percentage (%) for nominal values (Table 1). The statistical significance of the parameters was determined with a *p*-value < 0.05 using ANOVA for parametric, Kruskal–Wallis tests for non-parametric, and Chi-square tests for nominal variables. Additionally, post-hoc tests (pairwise *t*-test/Dunnett’s) were applied for group comparisons (Supplementary Table S2).

The cardiovascular risk scores of the participants from each study group were calculated based on the Framingham Heart Study [24]. A representative figure can be found in Supplementary Figure S1.

Metabolomics data were log-transformed to guarantee a normal distribution. A supervised multivariate regression orthogonal partial least square discriminant analysis (OPLS-DA) was used to separate orthogonal components that did not distinguish between the preset classes of samples from those that did. The metabolites with a variable importance of projection (VIP) score higher than 1 were considered significant for class differentiation (Supplementary Table S3). Multivariate analyses, including PCA and orthogonal partial least square discriminant analysis (OPLS-DA), were performed using the software SIMCA^®^ (version 16.0.1).

R version 4.2.1 was used to analyze the clinical data and to obtain linear models to identify significant metabolites (*p* < 0.05) differentiating the study groups (control (0) − HC (1) − HC + complications (2) − CAD (3), denoting disease progression) (Supplementary Table S4). The model also included the following confounders: age, gender, body mass index, and principal components 1 and 2. Additionally, a similar model was constructed without correcting for age to assess whether a significant effect of age was present (Supplementary Table S5). The multiple testing correction method (false discovery rate (FDR)) was used to adjust the nominal *p*-values. FDR < 0.1 was considered statistically significant.

## 3. Results

### 3.1. Characteristics of Participants

A total of 221 participants comprised the control (*n* = 40), HC (*n* = 35), HC + complications (*n* = 114), and CAD (*n* = 32) groups. The ages of all participants were between 40 and 74 years old. On average, the individuals in the control and hypercholesterolemia (HC) groups were slightly younger compared to those in the HC + complications and the coronary artery disease (CAD) groups. Given the illnesses impacting these groups, such as hypertension, diabetes, and atherosclerosis, it is typical for older individuals to have a greater risk of developing coronary artery disease (CAD). Data on the comparisons of each group can be found in Supplementary Table S2. The age difference between the control group and the HC group was not statistically significant. This finding was also observed in the HC group with complications compared to the CAD group (Supplementary Table S2). Pulse wave velocity (PWV) measurements reveal arterial stiffness. Values between 10 and 12 m/s are regarded as borderline, whereas measurements greater than 12 m/s suggest stiffness. Given that diabetic and hypertensive patients are at risk of developing atherosclerotic illnesses and CAD patients already have arterial stiffness, relatively higher readings in the HC + complications and CAD groups were anticipated. All groups were found to be overweight, with only a minor difference between the control and illness states, according to the body mass index (BMI) values that were higher than 25 kg/m^2^. Blood lipid, HbA1c, and BP levels were significantly higher in the disease groups compared to healthy controls, confirming the relevant disease states. Although the distributions of white blood cell (WBC) and red blood cell (RBC) counts and ALT levels between groups also had significant *p*-values, the values fell within the normal reference ranges of 4.5–5.5 × 10^6^/μL, 4–10 × 10^3^/μL, and 0–40 U/L respectively. The measured INR, CRP, liver, and kidney function parameters did not vary significantly between the groups (Table 1).

### 3.2. Multivariate Analysis

**Figure 1:**
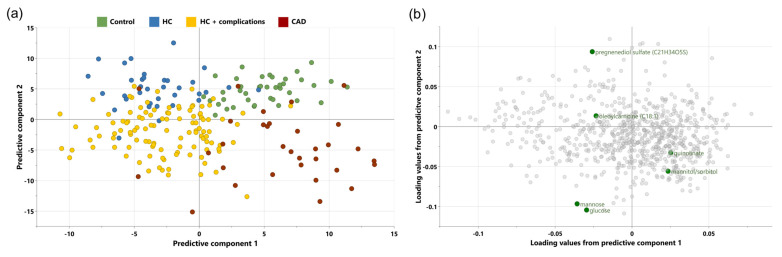
OPLS-DA of study groups. (**a**) The score plot graph shows principal component 1 on the *x*-axis versus principal component 2 on the *y*-axis. The analysis compared metabolites from control (green), HC (blue), HC + complications (yellow), and CAD (red) groups (R2Y = 47.4%, Q2 = 21.8%). (**b**)The loadings plot visually represents the metabolites that are associated with the four study groups in two dimensions.

Based on the OPLS-DA analysis, the metabolites were able to differentiate the study populations (Figure 1a). The R2Y value was 47.4% and Q2 was 21.8%. The metabolomic profiles differentiating the four study groups were effectively visualized through a two-dimensional scatterplot (Figure 1b). A total of 325 metabolites were identified as significantly separating the groups, with a VIP score greater than 1 (Supplementary Table S3).

### 3.3. Univariate Analysis

A linear regression model was used to demonstrate how variations in the ordered categorical groups influenced changes in metabolite levels. Our categorical groups presented a growing risk of atherosclerosis, such as healthy controls having a low risk, HC and HC + complications (high HbA1c and/or high BP) having a higher risk, and, finally, CAD patients certainly having the disease. The risk stratification of the study groups can be found in Supplementary Figure S1. The data were corrected for age, gender, BMI, and principal components 1 and 2 from the OPLS-DA model. Following *p*-value adjustment with multiple testing corrections, seven metabolites presented with a value of FDR < 0.1 (Table 2). The positive values in the regression model’s estimates indicate that the metabolite levels are expected to increase as the risk for CAD rises. Conversely, negative estimate values suggest that metabolite levels are expected to decrease as the risk of atherosclerosis becomes greater.

Mannitol/sorbitol (estimate: 0.447) and mannose (estimate: 0.171), which are metabolites within the fructose, mannose, and galactose metabolism sub-pathway, displayed notable increases in individuals with atherosclerosis. Similarly, glucose (estimate: 0.156), which is associated with carbohydrate metabolism, and ribitol (estimate: 0.137), within pentose metabolism, also exhibited positive associations with an increased risk of CAD.

In contrast, the decreased levels of pregnenediol sulfate (estimate: −0.314), from pregnenolone steroids, oleoylcarnitine (estimate: −0.150), from fatty acid metabolism, and quinolinate (estimate: −0.150), from nicotinate and nicotinamide metabolism, were observed when the CAD risk increased.

**Figure 2:**
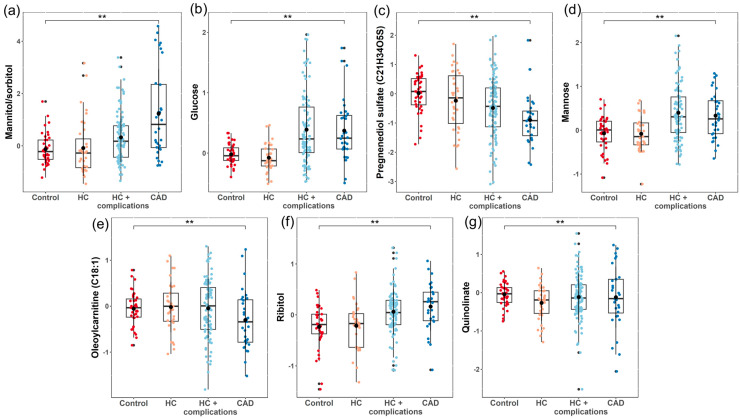
Differential measures of metabolites in the estimation of CAD. Boxplots show the FDR significant (<0.1) metabolites from a linear regression model. The levels of mannitol/sorbitol (**a**), glucose (**b**), mannose (**d**), and ribitol (**f**) increased from lower risk (controls) to higher risk (CAD) for atherosclerosis. On the other hand, the levels of pregnenediol sulfate (**c**), oleoylcarnitine (**e**), and quinolinate (**g**) decreased in the same manner. “**” represents FDR < 0.1.

The reported *p*-values and FDR values in Table 2 emphasize the robustness of these associations and the reliability of the observed changes in metabolite levels across the studied patient groups. Differential levels of metabolites associated with the increased risk of CAD are demonstrated in Figure 2. A similar analysis was performed without adjusting for age in the model and the results were similar, with comparable effect sizes and *p*-values for five out of six metabolites of interest (Supplementary Table S5).

### 3.4. Correlation Analysis

**Figure 3:**
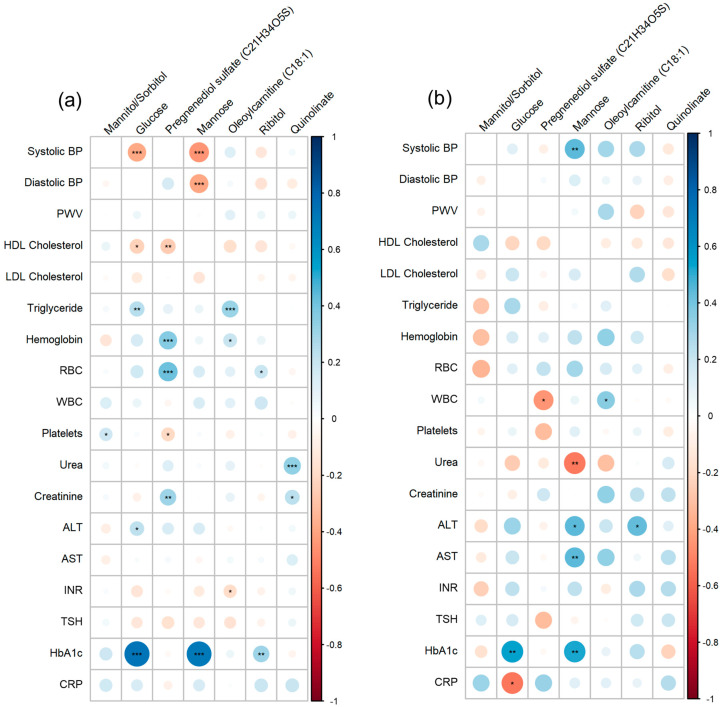
Correlations of metabolites and clinical parameters. The figures illustrate positive (blue) and negative (red) correlations between clinical parameters and metabolites in HC + complications (**a**) and CAD (**b**) groups. Statistically significant correlations were * *p* < 0.05, ** *p* < 0.01, *** *p* < 0.001.

To shed light on the potential mechanisms underlying the metabolic signature of CAD progression, the correlation between clinical traits and significant metabolites was analyzed in the HC + complications and CAD groups. Glucose and mannose were significantly positively correlated with HbA1c in both groups. Ribitol also showed a positive correlation with HbA1c, only in the HC + complications group. In the same group, HDL-C had a significant negative correlation with glucose and pregnenediol sulfate; however, this correlation was not significant in the CAD group (Figure 3).

## 4. Discussion

To elucidate the distinctive changes in metabolite profiles associated with the increasing risk of CAD and atherosclerosis, a comprehensive metabolomic analysis was conducted across four distinct groups: healthy controls, individuals with HC, individuals with HC and comorbid HT and/or diabetes, and individuals diagnosed with CAD. Utilizing state-of-the-art mass spectrometry-based metabolomics, we systematically quantified and compared the metabolite compositions of plasma samples obtained from each study group. The data were processed using advanced bioinformatics methods and identified seven metabolites that exhibited statistically significant alterations as the risk of CAD increased (Table 2 and Figure 2). Mannitol/sorbitol and mannose are derived from fructose, mannose, and galactose metabolism; glucose is sourced from glycolysis, gluconeogenesis, and pyruvate metabolism; pregnenediol sulfate (C21H34O5S) is obtained from pregnenolone steroids; oleoylcarnitine (C18:1) originates from fatty acid metabolism (acyl carnitine, monounsaturated); ribitol is a product of pentose metabolism; and quinolinate is formed within nicotinate and nicotinamide metabolism. These metabolites encompass a range of biochemical pathways, shedding light on the metabolic reprogramming that accompanies the development of atherosclerosis.

Inflammation and oxidative stress are major contributors to atherosclerosis. A large amount of cholesterol diffuses from the bloodstream to the intimal layer of the artery wall, where it is oxidized by free radicals. As a result, oxidized LDL (ox-LDL) accumulates in the artery wall, triggering the formation of atherosclerotic plaques [25]. Ox-LDL accumulation in the artery wall causes endothelial activation. It exacerbates the inflammatory response by producing adhesion molecules and chemokines, which help to recruit monocytes and T cells [26]. Monocytes, along with the lipid pool and necrotic cells, change into macrophages, ingest cholesterol, and differentiate into foam cells, creating the core of the atherosclerotic plaque [27]. Furthermore, growth factor and cytokine release mediate cell migration, proliferation, and collagen secretion, which altogether lead to atherosclerotic plaque formation [28]. Highlighting the primary players in atherosclerosis is critical because a considerable portion of the findings of this study, which will be discussed further, are related to inflammation and oxidative stress.

The monosaccharides fructose, mannose, and galactose are metabolized by the body. Fructose is found in honey and fruits, while mannose and galactose are present in smaller quantities in fruits and vegetables [29]. The escalating consumption of sugar is now considered a contributory factor in the worldwide increase in obesity, diabetes, and associated cardiometabolic risks [30]. Fructose metabolism, in particular, has been linked to a heightened risk of cardiometabolic diseases and a growing incidence of coronary artery disease (CAD) [31]. Research indicates that consuming high levels of fructose, especially when ingested with glucose, may trigger metabolic shifts in the liver, such as increased de novo lipogenesis (DNL), which can alter the lipid profiles in the blood and contribute to atherosclerosis [32]. Diets high in sucrose (exceeding 20% of total energy) are associated with raised plasma triglyceride levels, stemming from increased production by the liver and the reduced clearance of very low-density lipoprotein (VLDL) [33]. Additionally, galactose intake has been implicated in adverse cardiovascular effects [34]. Conversely, plasma mannose levels have been proposed as a new indicator for myocardial infarction and cardiovascular outcomes [35]. Our findings align with this evidence, showing a rise in mannose levels (estimate: 0.171) alongside an increased CAD risk. Furthermore, our research reveals a significant positive association between mannose levels and systolic blood pressure in CAD patients, suggesting that elevated mannose levels may enhance the risk of atherosclerotic vascular disease. On the other hand, it was stated that the association between mannose and myocardial infarction will not remain in case of blood glucose abnormalities [35]. Considering the elevated HbA1c levels in our complications and CAD groups, the predictive power of the mannose metabolite should be validated in future studies.

On the other hand, sorbitol and mannitol, classified as hexitols, are commonly utilized as low-calorie substitutes in sugar-free foods [36]. To date, there is no definitive evidence in the literature linking mannitol, sorbitol, or their combination to CAD. However, our study showed a clear rise in mannitol/sorbitol (estimate: 0.447) levels when the patient’s status evolved from healthy to CAD. This observation is pioneering and necessitates additional clinical research to confirm its validity.

In our study, we identified a significant link between elevated glucose levels and the risk of coronary artery disease (CAD), with an estimate of 0.156. The relationship between glucose and atherosclerosis, particularly in the context of CAD, occurs through complex mechanisms involving metabolic dysregulation and inflammation [37]. When blood sugar levels are high, there is an increase in the production of reactive oxygen species (ROS) and oxidative stress, which can damage vascular endothelial cells, making them more prone to inflammation and plaque formation [38]. Additionally, high glucose levels can cause the formation of advanced glycation end products (AGEs), which can contribute to inflammation and accumulate in blood vessel walls, promoting atherosclerosis [39,40]. Further, hyperglycemia induces insulin resistance, which also contributes to systemic inflammation and an increased risk of atherosclerosis [37]. This is because inflammatory cells migrate to the site of plaque formation, accelerating plaque growth [41]. Furthermore, hyperglycemia can impair the function of vascular endothelial cells, which are critical in maintaining proper blood vessel dilation, preventing blood clot formation, and regulating inflammation [42,43]. Hyperglycemia-induced platelet activation and aggregation may also contribute to the formation of blood clots within arteries, potentially leading to heart attacks or strokes [44].

Extensive studies have shown that elevated blood glucose levels can cause abnormalities in lipid metabolism, including higher levels of triglycerides and lower levels of HDL cholesterol [45]. These changes in lipid profiles are associated with an increased risk of CAD [46].

Pregnenolone sulfate is a type of sulfated steroid that has been studied for its effects on various receptors in the body, such as the inhibition of the GABAA receptor and the nicotinic acetylcholine receptor [47,48]. It has also been found to be a positive allosteric modulator of the *N*-methyl-d-aspartate receptor (NMDAR), which is associated with neurodevelopmental disorders [49]. Additionally, pregnenolone sulfate has been studied for its role in steroid production in the adrenal gland [50]. It is also a precursor for testosterone hormone synthesis [51]. Low levels of testosterone were found to be related to an increased risk for CAD [52,53]. Despite some studies showing a relationship between the serum levels of steroids and an increased risk of CAD [54,55,56], there was no established direct connection between pregnenediol sulfate and atherosclerotic vascular disease until recently.

In 2023, the EpiHealth study—a large-scale metabolomics investigation—in Sweden found that 37 metabolites were associated with incident CVD [57]. In comparison, out of these 37 metabolites, our regression analysis revealed 10 common metabolites with *p*-values less than 0.05 (Supplementary Table S4). Furthermore, the EpiHealth study showed that five metabolites improved CVD discrimination by 4%, when added to established cardiovascular disease risk variables, and it concluded that the metabolites may be incorporated into risk prediction scores in the future if replicated by others [57]. Pregnenediol sulfate is one of them, in which we found a significant reduction in its amount (estimate: −0.314) with the increased risk of CAD, showing the potential of this metabolite as a predictor of atherosclerosis.

Oleoylcarnitine (C18:1) is a type of acyl carnitine that is a product of the mitochondrial and peroxisomal processes of cellular energy metabolism. They are intermediates in fatty acid metabolism. A fatty acid moiety that might be long-chain, medium-chain, short-chain, branched, hydroxylated, saturated, or unsaturated and is attached to the biofactor L-carnitine in an acylcarnitine molecule. The body uses acyl carnitines, including oleoylcarnitine, to carry fatty acids into the mitochondria, where they are converted into energy [58]. The transport of short-, medium-, and long-carbon-chain acyl carnitines across the mitochondrial inner membrane in exchange for carnitine is carried out by the SLC25A20 transporter, also known as the carnitine acyl-carnitine carrier (CAC) [59].

Acyl carnitines are important diagnostic indicators for inborn defects of fatty acid oxidation because they play significant roles in several cellular energy metabolism pathways. They are being researched extensively as measures of energy metabolism, mitochondrial and peroxisomal oxidation activity deficiencies, insulin resistance, and physical activity. Acyl carnitines are becoming increasingly significant in metabolic investigations of various diseases, including metabolic disorders, cardiovascular diseases, diabetes, depression, neurologic disorders, and some malignancies [60].

Investigations are still ongoing to determine how acyl carnitines may contribute to the progression of CAD. However, current research indicates that acyl carnitines may have a substantial role in the onset and progression of cardiovascular disease [61]. Acyl carnitines are linked to the monitoring of macrophage FA catabolism and quantifying the status of oxidation [61]. Additionally, a study discovered that elevated serum even-chained acyl carnitines were linked, independently of conventional risk variables, to an increased risk of cardiovascular death and, to a lesser extent, acute myocardial infarction [62]. In a different investigation, it was discovered that patients with type 2 diabetes mellitus had greater plasma levels of certain acylcarnitine metabolites, which were linked to an increased risk of CVD [63]. Overall, these findings indicate acyl carnitines’ association with CAD.

In this study, we found a specific type of acyl carnitine, oleoylcarnitine (C18:1), to be reduced (estimate: −0.15) as the risk of CAD increased. Oleoylcarnitine is derived from oleic acid [60]. A number of studies have shown that oleic acid intake has an atheroprotective effect [64,65,66]. On the other hand, high plasma levels of oleic acid were found to be related to an increased risk of atherosclerosis [67]. In this context, there is a difference between the blood levels and dietary intake of oleic acid in terms of CAD risk. To our knowledge, no study has shown a direct association between oleoylcarnitine and CAD risk. Our findings suggest that oleoylcarnitine may serve as a promising indicator for the development of CAD, opening up new areas of investigation.

Further, our results showed that when the CAD risk was higher, ribitol levels were expected to rise (estimate: 0.137). A previous metabolomics study showed ribitol measures to be significantly reduced in CAD patients compared to healthy controls [68].

Ribitol is a sugar alcohol that is metabolized primarily through the pentose phosphate pathway (PPP), a major cellular pathway involved in the generation of NADPH (reducing agent) and ribose-5-phosphate (a precursor for nucleotide synthesis) [69]. Ribitol can enter the PPP and contribute to the production of NADP. NADPH is critical in combating oxidative stress and supporting various anabolic processes, including fatty acid and cholesterol synthesis [70]. Therefore, disruptions in NADPH production could potentially contribute to increased oxidative stress and inflammation, which are key contributors to the development and progression of atherosclerosis [71].

While the direct link between ribitol or pentose metabolism and atherosclerosis has not been extensively studied, disturbances in these metabolic pathways could impact cellular processes and potentially contribute to disease development. However, more research is needed to establish a direct connection between ribitol metabolism and atherosclerosis or CAD.

Quinolinate is an additional metabolite whose concentrations decreased (approximately −0.15) in our study as a result of the increased risk of CAD. Quinolinate, alternatively referred to as quinolinic acid, is generated via the kynurenine pathway during the metabolism of tryptophan. Quinolinate performs several noteworthy biological roles, such as facilitating energy metabolism and redox reactions via the kynurenine pathway, neuroactivation by acting as an agonist for the N-methyl-D-aspartate (NMDA) receptor, and the biosynthesis of nicotinamide adenine dinucleotide (NAD+) [72,73,74]. Furthermore, it may play a role in neurodegenerative diseases [75].

The role of quinolinate in atherosclerosis is indirect and less clear. The development of atherosclerosis could potentially be influenced by the chronic activation of the kynurenine pathway, which subsequently leads to inflammation [76,77]. Conversely, our findings demonstrate an explicit correlation between the advancement of CAD and quinolinate. The correlation between quinolinate and atherosclerosis necessitates additional investigation. This study had several limitations. One of these was the study model for disease progression. This observational study aimed to identify metabolites related to disease progression by analyzing four stages from health to CAD. The most suitable model would be a prospective study examining the disease progression of previously healthy individuals. However, knowing that hypercholesterolemia, diabetes, and hypertension are high risk factors for CAD development, we believe that our study provides information about the disease’s progression in stages.

The second limitation of this study was the small sample size. Larger groups of participants would produce more generalizable results. Although the participant counts were relatively small, the number of metabolites was large and quite comprehensive, giving a novel aspect to our findings.

The third limitation was that there were significant age differences between the control and the HC with complications and CAD groups. This variance was an unintended consequence of the available samples in the Qatar BioBank. Given the nature of these diseases, it is rare to observe healthy controls and similarly younger individuals with CAD, diabetes, or HT.

The next limitation was the absence of diagnostic measures of the CAD group. Diagnostic validation would enhance the reliability of the data and overall strength of the conclusions drawn. The metabolomics data were obtained from the Qatar BioBank and clinical test results validating the diagnosis were not available in their database. Relying solely on self-reported data can introduce biases, as patients may not always accurately report their health status. However, the medications that the CAD patients used clearly supported their diagnosis, such as beta blockers, aspirin, platelet aggregation inhibitors, calcium channel blockers, angiotensin-converting enzyme inhibitors, angiotensin receptor blockers, and cholesterol-lowering medications. The list of medications can be found in Supplementary Table S6.

The final limitation was the potential influence of confounding factors, including medication use among participants, the duration of complications, or the presence of silent atherosclerotic vascular disease in the study groups. The presence of these confounders could impact the results, such as controls exhibiting a disease state or the disease groups experiencing the anti-inflammatory effects of statins. To enhance the reliability of our results, we adjusted for principal components from PCA, which captured some of the variations related to unknown confounders such as medication, diet, lifestyle, etc. However, recognizing the complexity of these influences, we acknowledge the need for more comprehensive and controlled future studies that specifically target and explore these potential confounding elements.

Atherosclerotic vascular disease prognosis is significantly aided by the results of this investigation. However, larger sample sizes and clinical data that support the diagnosis of atherosclerosis are required in future studies.

## 5. Conclusions

As CAD progresses from a healthy state to one complicated by hypercholesterolemia (HC), hypertension (HT), diabetes, and ultimately CAD itself, there is an observable rise in the levels of glucose, mannitol/sorbitol, and ribitol. This underscores the disruption of carbohydrate metabolism as a pivotal factor in CAD. Additionally, the observed decline in pregnenediol sulfate and oleoylcarnitine levels may point to shifts in lipid metabolism, particularly concerning acyl carnitine and monounsaturated fatty acids, during the evolution of CAD. The significant decline in quinolinate, which is associated with the metabolism of nicotine and nicotinamide, provides additional evidence for the interplay between various metabolic pathways and their contributions to the pathogenesis of atherosclerosis. These revelations augment our understanding of the metabolic dysregulations that contribute to atherosclerosis and could potentially direct subsequent investigations and the development of precise treatments aimed at reducing cardiovascular risks.

## Figures and Tables

**Table 1:** Characteristics of the study groups. The table represents clinical measurement data of control, HC, HC + complications, and CAD groups. The values are presented as mean (SD), median (IQR), and percentage (%), based on the parametric, non-parametric, and nominal variables, respectively. Differences between the groups were analyzed using ANOVA for parametric, Kruskal–Wallis for non-parametric, and Chi-square test for nominal variables. A *p*-value significance level of 0.05 was used.

Variable	Control	HC	HC + Complications	CAD	*p*-Value
*n*	40	35	114	32	
Gender					0.475
Male,	18 (45%),	16 (45.71%),	63 (55.26%),	19 (59.38%),	
Female	22 (55)	19 (54.29%)	51 (44.74%)	13 (40.63%)	
Age	44 (42–46.25)	45 (42–47)	51 (45–56)	54.5 (49–59)	<0.001
PWV m/s	10.35 (9.4–11.1)	11.8 (10.3–12.8)	14.05 (11.9–17.7)	13.6 (11.2–19.35)	<0.001
BMI (kg/m^2^)	28.01 (4.45)	27.82 (3.89)	31.01 (5.36)	31.5 (5.27)	<0.001
Systolic BP (mmHg)	108 (101–116.25)	115 (106–124.5)	133 (120.75–141.75)	123 (118.25–129.5)	<0.001
Diastolic BP (mmHg)	71.45 (6.00)	75.37 (8.45)	83.87 (12.05)	76.81 (10.82)	<0.001
Hemoglobin (g/dL)	13.01 (2.52)	13.5 (1.87)	13.96 (1.83)	13.56 (1.59)	0.062
RBC (×10^6^/μL)	4.84 (0.66)	4.81 (0.53)	5.06 (0.54)	4.89 (0.56)	0.042
WBC (×10^3^/μL)	6.25 (5.47–7.3)	6 (5.3–7.62)	6.8 (6–8.4)	7.6 (6.25–8.87)	0.005
Platelets (×10^3^/μL)	245 (197.25–300.75)	254.5 (213.25–283.5)	234 (196–279)	219.5 (192.25–273.25)	0.471
Urea (mmol/L)	4.5 (3.77–5.57)	4.5 (3.7–5.05)	4.5 (3.7–5.37)	5 (4.3–6.17)	0.157
Creatinine (μmol/L)	64.5 (55.50–76.25)	64 (56.5–78.5)	69.5 (58–80)	72.5 (62–83.5)	0.226
ALT (U/L)	17 (14.75–22.5)	22 (15–32.5)	22 (17–31)	21.5 (17.5–27.75)	0.024
AST (U/L)	16 (15–20.25)	19 (16.5–24)	18 (15–21)	18 (15–22)	0.298
HDL Cholesterol (mmol/L)	1.40 (1.24–1.57)	1.28 (1.11–1.49)	1.18 (1.00–1.40)	1.185 (0.975–1.51)	0.009
LDL Cholesterol (mmol/L)	2.84 (2–3)	4 (3.88–4.60)	4 (3.64–4.37)	2.42 (1.98–3.12)	<0.001
Triglyceride (mmol/L)	1.04 (0.81–1.32)	1.9 (1.24–2.54)	1.93 (1.44–2.58)	1.5 (0.99–2.10)	<0.001
INR	1 (1–1.1)	1 (1–1.1)	1 (1–1.1)	1 (1–1.1)	0.458
TSH (mIU/L)	1.27 (1.05–1.86)	1.64 (1.06–2.67)	1.33 (0.90–1.94)	1.44 (0.90–2.30)	0.268
HbA1c %	5.4 (5.1–5.5)	5.5 (5.15–5.8)	6.5 (5.8–8.07)	6.3 (5.7–7.17)	<0.001
CRP (mg/L)	5 (5–6)	5 (5–7.5)	5 (5–8)	5 (5–9)	0.721

**Table 2:** The metabolites that are associated with CAD progression. The table shows the metabolites that are significantly associated with the risk of atherosclerosis. Estimate values show the predicted increase (+) or decrease (−) in the metabolite levels while the risk for atherosclerosis rises. A linear regression model was used. Significant values are those with a *p*-value < 0.05 and FDR < 0.1.

Metabolites	Sub-Pathway	Super-Pathway	Estimate	SE	*p*-Value	FDR
Mannitol/Sorbitol	Fructose, Mannose, and Galactose Metabolism	Carbohydrate	0.447	0.097	<0.001	0.005
Glucose	Glycolysis, Gluconeogenesis, and Pyruvate Metabolism	Carbohydrate	0.156	0.041	<0.001	0.062
Pregnenediol Sulfate (C21H34O5S)	Pregnenolone Steroids	Lipid	−0.314	0.084	<0.001	0.062
Mannose	Fructose, Mannose, and Galactose Metabolism	Carbohydrate	0.171	0.048	<0.001	0.078
Oleoylcarnitine (C18:1)	Fatty Acid Metabolism (Acyl Carnitine, Monounsaturated)	Lipid	−0.150	0.042	<0.001	0.078
Ribitol	Pentose Metabolism	Carbohydrate	0.137	0.040	<0.001	0.092
Quinolinate	Nicotinate and Nicotinamide Metabolism	Cofactors and Vitamins	−0.150	0.044	<0.001	0.092

## Data Availability

Restrictions apply to the availability of these data. Data were obtained from Qatar Biobank (https://www.qatarbiobank.org.qa) under a confidentiality agreement with Qatar University (accessed on 4 August 2022). Supplementary data are freely available.

## Supplementary Materials

The following supporting information can be downloaded at: https://www.mdpi.com/article/10.3390/metabo14060292/s1, Supplementary Table S1: Metabolomics data of the study groups. Supplementary Table S2: Demographic and clinical characteristics of the participants. Supplementary Table S3: Metabolites that are significant for class differentiation. Supplementary Table S4: Linear regression analysis. Supplementary Table S5: Linear regression analysis without correcting for age. Supplementary Table S6: List of medications used by the disease groups. Supplementary Figure S1: Comparison of the study groups with the cardiovascular risk groups.

## Abbreviations

HC: Hypercholesterolemia, CAD: Coronary Artery Disease, CVD: Cardiovascular Disease, PWV: Pulse Wave Velocity, BMI: Body Mass Index, BP: Blood Pressure, RBC: Red Blood Cell, WBC: White Blood Cell, ALT: Alanine Aminotransferase, AST: Aspartate Aminotransferase, HDL: High-Density Lipoprotein, LDL: Low-Density Lipoprotein, INR: International Normalization Ratio, TSH: Thyroid Stimulating Hormone, HbA1c: Hemoglobin A1c, CRP: C-Reactive Protein.
