# Supplementary material for: Metabolomics Profiling of Stages of Coronary Artery Disease Progression
Source: Metabolites. 2024 May 22;14(6):292. doi: 10.3390/metabo14060292 (PMC11205943; doi:10.3390/metabo14060292)
Supplement: Supplementary file 1 [file metabolites-14-00292-s001.zip › Figure S1- CV risk assessment.pdf]

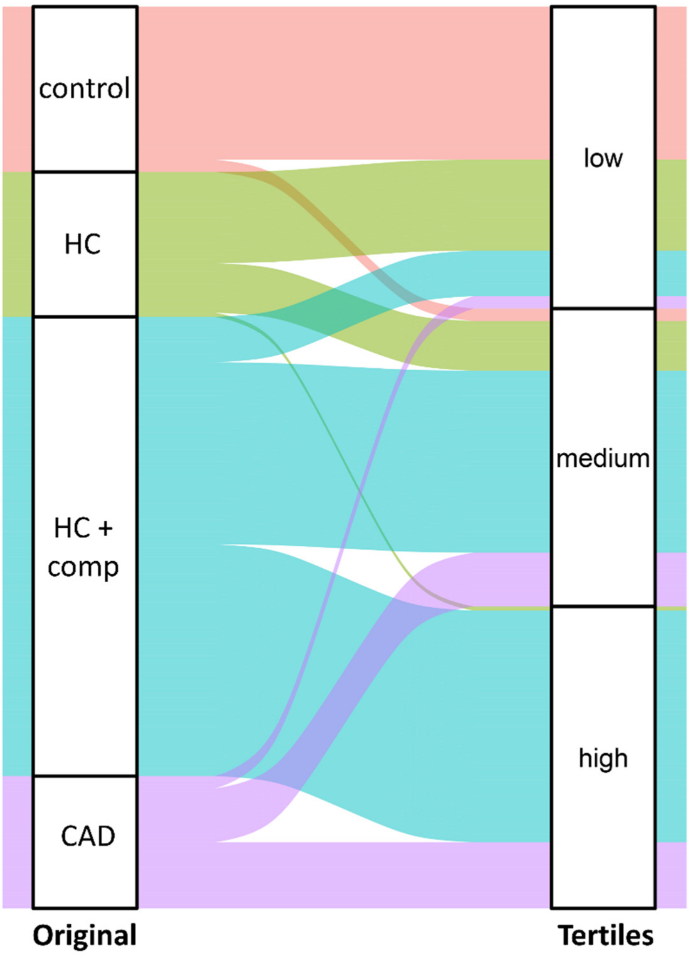

Figure S1. Comparison of the study groups with the cardiovascular risk groups. Sankey diagram representing the difference in the proportion of participants between the original stratification and tertiles calculated from cardiovascular risk assessment. Cardiovascular risk assessment was performed using the formula:

"Risk_Factors = (ln(Age) × 3.06117) + (ln(Total_cholesterol) × 1.12370) − (ln(HDL_cholesterol) × 0.93263) + (ln(Systolic_blood_pressure) × On_blood_pressure_medication) + Cigarette_smoker + Diabetes_present - 23.9802.

Risk = 100 × (1 - $0.88936^{e(\text{Risk\_Factors})}$)"

Calculated risk score <10% labelled as "low", 10-20% as "medium" and >20% as "high" referring the low, medium and high risks respectively.
