# Supplementary material for: Metabolomics Profiling of Stages of Coronary Artery Disease Progression
Source: Metabolites. 2024 May 22;14(6):292. doi: 10.3390/metabo14060292 (PMC11205943; doi:10.3390/metabo14060292)
Supplement: Supplementary file 1 [file metabolites-14-00292-s001.zip › Table S2-Patient demographics.pdf]

Table S2. Demographic and clinical characteristics of the participants. The table represents clinical measurement data of control, HC, HC+complications, and CAD groups. The values are presented as mean (SD), median (IQR), and percentage (%), based on the parametric, non-parametric, and nominal variables respectively. Differences between the groups were analyzed using ANOVA for parametric, Kruskal Wallis for non-parametric, and Chi-square test for nominal variables. Post-HOC tests (Pairwise T-test/Dunnett's) were applied for group comparisons. A *p*-value significance level of 0.05 was used.

**Variable**	Control	High Cholesterol (HC)	HC + complications	CAD	anova /kruskal	control vs HC	control vs HC+complications	control vs CAD	HC vs HC + complications	HC vs CAD	HC + complications vs CAD
n	40	35	114	32							
Gender (M: Male, F: Female)	1:18 (45%), 2:22 (55)	1:16 (45.71%), 2: 19 (54.29%)	1: 63 (55.26%), 2: 51 (44.74%)	1: 19 (59.38%), 2: 13 (40.63%)	0.475	0.864	0.35	0.329	0.425	0.382	0.831
Age	44 (42-46.25)	45 (42-47)	51 (45-56)	54.5 (49-59)	<0.001	0.549	<0.001	<0.001	<0.001	<0.001	0.251
PWV m/s	10.35 (9.4-11.1)	11.8 (10.3-12.8)	14.05 (11.9-17.7)	13.6 (11.2-19.35)	<0.001	0.004	<0.001	<0.001	<0.001	0.008	0.697
BMI (kg/m^2)	28.01 (4.45)	27.82 (3.89)	31.01 (5.36)	31.5 (5.27)	<0.001	0.998	0.007	0.017	0.006	0.014	0.953
Systolic BP (mmHg)	108 (101-116.25)	115 (106-124.5)	133 (120.75-141.75)	123 (118.25-129.5)	<0.001	0.063	<0.001	<0.001	<0.001	0.02	0.05
Diastolic BP (mmHg)	71.45 (6.00)	75.37 (8.45)	83.87 (12.05)	76.81 (10.82)	<0.001	0.372	<0.001	0.139	<0.001	0.943	0.005
Hemoglobin (g/dL)	13.01 (2.52)	13.5 (1.87)	13.96 (1.83)	13.56 (1.59)	0.062	0.7	0.042	0.627	0.625	0.999	0.741
RBC (x10^6/uL)	4.84 (0.66)	4.81 (0.53)	5.06 (0.54)	4.89 (0.56)	0.042	0.997	0.142	0.984	0.115	0.954	0.41
WBC (x10^3/uL)	6.25 (5.47-7.3)	6 (5.3-7.62)	6.8 (6-8.4)	7.6 (6.25-8.87)	0.005	0.863	0.038	0.021	0.038	0.021	0.302
Platelets (x10^3/uL)	245 (197.25-300.75)	254.5 (213.25-283.5)	234 (196-279)	219.5 (192.25-273.25)	0.471	0.723	0.621	0.604	0.604	0.604	0.621

Urea (mmol/L)	4.5 (3.77-5.57)	4.5 (3.7-5.05)	4.5 (3.7-5.37)	5 (4.3-6.17)	0.157	0.421	0.835	0.341	0.421	0.158	0.208
Creatinine (umol/L)	64.5 (55.50-76.25)	64 (56.5-78.5)	69.5 (58-80)	72.5 (62-83.5)	0.226	0.726	0.367	0.339	0.477	0.367	0.448
ALT (U/L)	17 (14.75-22.5)	22 (15-32.5)	22 (17-31)	21.5 (17.5-27.75)	0.024	0.121	0.013	0.121	0.765	0.871	0.794
AST (U/L)	16 (15-20.25)	19 (16.5-24)	18 (15-21)	18 (15-22)	0.298	0.344	0.411	0.411	0.411	0.555	0.874
HDL Cholesterol (mmol/L)	1.40 (1.24-1.57)	1.28 (1.11-1.49)	1.18 (1.00-1.40)	1.185 (0.975-1.51)	0.009	0.543	0.013	0.086	0.086	0.316	0.67
LDL Cholesterol (mmol/L)	2.84 (2-3)	4 (3.88-4.60)	4 (3.64-4.37)	2.42 (1.98-3.12)	<0.001	<0.001	<0.001	0.394	0.351	<0.001	<0.001
Triglyceride (mmol/L)	1.04 (0.81-1.32)	1.9 (1.24-2.54)	1.93 (1.44-2.58)	1.5 (0.99-2.10)	<0.001	<0.001	<0.001	0.035	0.512	0.116	0.015
INR	1 (1-1.1)	1 (1-1)	1 (1-1.1)	1 (1-1.1)	0.458	0.566	0.566	0.676	0.566	0.566	0.763
TSH (mIU/L)	1.27 (1.05-1.86)	1.64 (1.06-2.67)	1.33 (0.90-1.94)	1.44 (0.90-2.30)	0.268	0.403	0.915	0.915	0.298	0.41	0.915
HbA1c %	5.4 (5.1-5.5)	5.5 (5.15-5.8)	6.5 (5.8-8.07)	6.3 (5.7-7.17)	<0.001	0.323	<0.001	<0.001	<0.001	<0.001	0.49
CRP (mg/L)	5 (5-6)	5 (5-7.5)	5 (5-8)	5 (5-9)	0.721	0.751	0.751	0.751	0.751	0.977	0.751
