# Supplementary material for: Metabolomics Profiling of Stages of Coronary Artery Disease Progression
Source: Metabolites. 2024 May 22;14(6):292. doi: 10.3390/metabo14060292 (PMC11205943; doi:10.3390/metabo14060292)
Supplement: Supplementary file 1 [file metabolites-14-00292-s001.zip › Table S3- VIP list of metabolites.pdf]

Table S3. Metabolites that are significant for class differentiation. The table shows the metabolites with variable importance of projection score higher than 1(VIP> 1).

BIOCHEMICAL	Super pathway	Sub pathway	M2.VIP[3+2+0]
cholesterol	Lipid	Sterol	2.087
behenoyl sphingomyelin (d18:1/22:0)	Lipid	Sphingomyelins	1.935
sphingomyelin (d18:1/20:0, d16:1/22:0)	Lipid	Sphingomyelins	1.854
glucose	Carbohydrate	Glycolysis, Gluconeogenesis, and Pyruvate Metabolism	1.820
sphingomyelin (d18:1/21:0, d17:1/22:0, d16:1/23:0)	Lipid	Sphingomyelins	1.786
pregnenediol sulfate (C21H34O5S)	Lipid	Pregnenolone Steroids	1.723
sphingomyelin (d18:2/16:0, d18:1/16:1)	Lipid	Sphingomyelins	1.718
sphingomyelin (d18:1/22:1, d18:2/22:0, d16:1/24:1)	Lipid	Sphingomyelins	1.711
2-hydroxybutyrate/2-hydroxyisobutyrate	Amino Acid	Glutathione Metabolism	1.708
N-palmitoyl-sphingosine (d18:1/16:0)	Lipid	Ceramides	1.698
pregnenetriol sulfate	Lipid	Pregnenolone Steroids	1.697
1-linoleoyl-GPC (18:2)	Lipid	Lysophospholipid	1.692
N-stearoyl-sphingosine (d18:1/18:0)	Lipid	Ceramides	1.684
1-palmitoyl-GPE (16:0)	Lipid	Lysophospholipid	1.676
1-stearoyl-GPE (18:0)	Lipid	Lysophospholipid	1.670
3-methyl-2-oxobutyrate	Amino Acid	Leucine, Isoleucine and Valine Metabolism	1.668
1,5-anhydroglucitol (1,5-AG)	Carbohydrate	Glycolysis, Gluconeogenesis, and Pyruvate Metabolism	1.667
sphingomyelin (d18:2/23:0, d18:1/23:1, d17:1/24:1)	Lipid	Sphingomyelins	1.664
sphingomyelin (d17:1/16:0, d18:1/15:0, d16:1/17:0)	Lipid	Sphingomyelins	1.653
sphingomyelin (d18:1/14:0, d16:1/16:0)	Lipid	Sphingomyelins	1.640
erythronate	Carbohydrate	Aminosugar Metabolism	1.634
mannose	Carbohydrate	Fructose, Mannose and Galactose Metabolism	1.630
1-stearoyl-GPC (18:0)	Lipid	Lysophospholipid	1.624
3-methyl-2-oxovalerate	Amino Acid	Leucine, Isoleucine and Valine Metabolism	1.623
5alpha-pregnan-3beta,20beta-diol monosulfate (1)	Lipid	Progestin Steroids	1.619
2,3-dihydroxy-5-methylthio-4-pentenoate (DMTPA)	Amino Acid	Methionine, Cysteine, SAM and Taurine Metabolism	1.618
palmitoyl sphingomyelin (d18:1/16:0)	Lipid	Sphingomyelins	1.617
sphingomyelin (d18:1/19:0, d19:1/18:0)	Lipid	Sphingomyelins	1.600
mannonate	Xenobiotics	Food Component/Plant	1.594
1,2-dilinoleoyl-GPC (18:2/18:2)	Lipid	Phosphatidylcholine (PC)	1.593
glycosyl ceramide (d18:1/20:0, d16:1/22:0)	Lipid	Hexosylceramides (HCER)	1.590

BIOCHEMICAL	Super pathway	Sub pathway	M2.VIP[3+2+0]
1-palmitoyl-2-docosahexaenoyl-GPE (16:0/22:6)	Lipid	Phosphatidylethanolamine (PE)	1.590
leucine	Amino Acid	Leucine, Isoleucine and Valine Metabolism	1.590
4-methyl-2-oxopentanoate	Amino Acid	Leucine, Isoleucine and Valine Metabolism	1.570
1-palmitoyl-GPC (16:0)	Lipid	Lysophospholipid	1.569
sphingomyelin (d18:1/17:0, d17:1/18:0, d19:1/16:0)	Lipid	Sphingomyelins	1.558
sphingomyelin (d18:0/20:0, d16:0/22:0)	Lipid	Dihydrosphingomyelins	1.539
sphingomyelin (d18:2/18:1)	Lipid	Sphingomyelins	1.533
oleoyl-linoleoyl-glycerol (18:1/18:2) [1]	Lipid	Diacylglycerol	1.533
1-palmitoyl-2-arachidonoyl-GPE (16:0/20:4)	Lipid	Phosphatidylethanolamine (PE)	1.532
pregnanolone/allopregnanolone sulfate	Lipid	Progestin Steroids	1.527
valine	Amino Acid	Leucine, Isoleucine and Valine Metabolism	1.527
5alpha-pregnan-3beta,20alpha-diol disulfate	Lipid	Progestin Steroids	1.516
palmitoyl dihydrosphingomyelin (d18:0/16:0)	Lipid	Dihydrosphingomyelins	1.514
5alpha-pregnan-3beta,20alpha-diol monosulfate (2)	Lipid	Progestin Steroids	1.511
pregnenediol disulfate (C21H34O8S2)	Lipid	Pregnenolone Steroids	1.503
sphingomyelin (d18:2/21:0, d16:2/23:0)	Lipid	Sphingomyelins	1.498
isoleucine	Amino Acid	Leucine, Isoleucine and Valine Metabolism	1.495
N2,N5-diacetylornithine	Amino Acid	Urea cycle; Arginine and Proline Metabolism	1.488
androstenediol (3alpha, 17alpha) monosulfate (2)	Lipid	Androgenic Steroids	1.482
sphingomyelin (d18:1/24:1, d18:2/24:0)	Lipid	Sphingomyelins	1.475
methylsuccinoylcarnitine	Amino Acid	Leucine, Isoleucine and Valine Metabolism	1.474
hydroxyasparagine	Amino Acid	Alanine and Aspartate Metabolism	1.473
dehydroepiandrosterone sulfate (DHEA-S)	Lipid	Androgenic Steroids	1.473
1-linoleoyl-2-arachidonoyl-GPC (18:2/20:4n6)	Lipid	Phosphatidylcholine (PC)	1.468
1-palmitoyl-2-linoleoyl-GPE (16:0/18:2)	Lipid	Phosphatidylethanolamine (PE)	1.467
linoleoyl-linoleoyl-glycerol (18:2/18:2) [1]	Lipid	Diacylglycerol	1.467
pregnenolone sulfate	Lipid	Pregnenolone Steroids	1.466
stearoyl sphingomyelin (d18:1/18:0)	Lipid	Sphingomyelins	1.464
5-methylthioadenosine (MTA)	Amino Acid	Polyamine Metabolism	1.461
gamma-glutamylthreonine	Peptide	Gamma-glutamyl Amino Acid	1.460

BIOCHEMICAL	Super pathway	Sub pathway	M2.VIP[3+2+0]
2-O-methylascorbic acid	Cofactors and Vitamins	Ascorbate and Aldarate Metabolism	1.459
1,2-dipalmitoyl-GPC (16:0/16:0)	Lipid	Phosphatidylcholine (PC)	1.459
hydroxypalmitoyl sphingomyelin (d18:1/16:0(OH))	Lipid	Sphingomyelins	1.458
1-(1-enyl-palmitoyl)-2-linoleoyl-GPC (P-16:0/18:2)	Lipid	Plasmalogen	1.452
gamma-glutamylisoleucine	Peptide	Gamma-glutamyl Amino Acid	1.448
2-aminobutyrate	Amino Acid	Glutathione Metabolism	1.447
4-cholesten-3-one	Lipid	Sterol	1.441
androstenediol (3beta,17beta) monosulfate (1)	Lipid	Androgenic Steroids	1.437
5-(galactosylhydroxy)-L-lysine	Amino Acid	Lysine Metabolism	1.436
1-palmitoyl-2-oleoyl-GPC (16:0/18:1)	Lipid	Phosphatidylcholine (PC)	1.435
alpha-tocopherol	Cofactors and Vitamins	Tocopherol Metabolism	1.434
N-palmitoyl-sphingadienine (d18:2/16:0)	Lipid	Ceramides	1.434
palmitoyl-sphingosine-phosphoethanolamine (d18:1/16:0)	Lipid	Ceramide PEs	1.433
alpha-hydroxyisocaproate	Amino Acid	Leucine, Isoleucine and Valine Metabolism	1.432
1-stearoyl-2-oleoyl-GPC (18:0/18:1)	Lipid	Phosphatidylcholine (PC)	1.431
5alpha-pregnan-diol disulfate	Lipid	Progestin Steroids	1.421
erythritol	Xenobiotics	Food Component/Plant	1.418
fructose	Carbohydrate	Fructose, Mannose and Galactose Metabolism	1.417
oleoyl-linoleoyl-glycerol (18:1/18:2) [2]	Lipid	Diacylglycerol	1.411
glycosyl-N-palmitoyl-sphingosine (d18:1/16:0)	Lipid	Hexosylceramides (HCER)	1.410
1-oleoyl-GPC (18:1)	Lipid	Lysophospholipid	1.406
sphingomyelin (d18:1/18:1, d18:2/18:0)	Lipid	Sphingomyelins	1.403
pregnenetriol disulfate	Lipid	Pregnenolone Steroids	1.401
glycerophosphoethanolamine	Lipid	Phospholipid Metabolism	1.400
21-hydroxypregnenolone disulfate	Lipid	Pregnenolone Steroids	1.398
N1-methylinosine	Nucleotide	Purine Metabolism, (Hypo)Xanthine/Inosine containing	1.389
adenine	Nucleotide	Purine Metabolism, Adenine containing	1.385
gluconate	Xenobiotics	Food Component/Plant	1.380
1-palmitoyl-2-oleoyl-GPE (16:0/18:1)	Lipid	Phosphatidylethanolamine (PE)	1.379
1-(1-enyl-palmitoyl)-2-palmitoyl-GPC (P-16:0/16:0)	Lipid	Plasmalogen	1.374
2-aminoadipate	Amino Acid	Lysine Metabolism	1.371
1-palmitoyl-2-dihomo-linolenoyl-GPC (16:0/20:3n3 or 6)	Lipid	Phosphatidylcholine (PC)	1.371

BIOCHEMICAL	Super pathway	Sub pathway	M2.VIP[3+2+0]
androstenediol (3beta,17beta) disulfate (2)	Lipid	Androgenic Steroids	1.361
1-stearoyl-2-oleoyl-GPE (18:0/18:1)	Lipid	Phosphatidylethanolamine (PE)	1.347
1-arachidonoyl-GPE (20:4n6)	Lipid	Lysophospholipid	1.347
1-methyl-4-imidazoleacetate	Amino Acid	Histidine Metabolism	1.344
homocitrulline	Amino Acid	Urea cycle; Arginine and Proline Metabolism	1.340
arabitol/xylitol	Carbohydrate	Pentose Metabolism	1.337
sphingomyelin (d18:0/18:0, d19:0/17:0)	Lipid	Dihydrosphingomyelins	1.336
GlcNAc sulfate conjugate of C21H34O2 steroid	Partially Characterized Molecules	Partially Characterized Molecules	1.334
gamma-glutamylvaline	Peptide	Gamma-glutamyl Amino Acid	1.331
N6-methyladenosine	Nucleotide	Purine Metabolism, Adenine containing	1.330
sphingomyelin (d18:2/14:0, d18:1/14:1)	Lipid	Sphingomyelins	1.329
androstenediol (3alpha, 17alpha) monosulfate (3)	Lipid	Androgenic Steroids	1.323
1-palmitoyl-2-arachidonoyl-GPC (16:0/20:4n6)	Lipid	Phosphatidylcholine (PC)	1.318
gamma-glutamylleucine	Peptide	Gamma-glutamyl Amino Acid	1.318
guaiacol sulfate	Xenobiotics	Benzoate Metabolism	1.317
cerotoylcarnitine (C26)	Lipid	Fatty Acid Metabolism (Acyl Carnitine, Long Chain Saturated)	1.317
glutamate	Amino Acid	Glutamate Metabolism	1.316
sphingomyelin (d17:1/14:0, d16:1/15:0)	Lipid	Sphingomyelins	1.313
1-lignoceroyl-GPC (24:0)	Lipid	Lysophospholipid	1.311
isovalerylcarnitine (C5)	Amino Acid	Leucine, Isoleucine and Valine Metabolism	1.309
sphingomyelin (d18:1/20:1, d18:2/20:0)	Lipid	Sphingomyelins	1.309
pseudouridine	Nucleotide	Pyrimidine Metabolism, Uracil containing	1.307
myristoyl dihydrosphingomyelin (d18:0/14:0)	Lipid	Dihydrosphingomyelins	1.301
glycosyl-N-stearoyl-sphingosine (d18:1/18:0)	Lipid	Hexosylceramides (HCER)	1.298
hydroxy-N6,N6,N6-trimethyllysine	Amino Acid	Lysine Metabolism	1.295
sphingomyelin (d17:2/16:0, d18:2/15:0)	Lipid	Sphingomyelins	1.293
methionine sulfone	Amino Acid	Methionine, Cysteine, SAM and Taurine Metabolism	1.292
1-linoleoyl-GPE (18:2)	Lipid	Lysophospholipid	1.291
metabolonic lactone sulfate	Partially Characterized Molecules	Partially Characterized Molecules	1.289
fructosyllysine	Amino Acid	Lysine Metabolism	1.288
4-hydroxyphenylacetylglutamine	Peptide	Acetylated Peptides	1.284

BIOCHEMICAL	Super pathway	Sub pathway	M2.VIP[3+2+0]
N2,N2-dimethylguanosine	Nucleotide	Purine Metabolism, Guanine containing	1.280
1-ribosyl-imidazoleacetate	Amino Acid	Histidine Metabolism	1.278
androstenediol (3beta,17beta) disulfate (1)	Lipid	Androgenic Steroids	1.278
sphingomyelin (d18:2/24:1, d18:1/24:2)	Lipid	Sphingomyelins	1.278
1-stearoyl-2-arachidonoyl-GPE (18:0/20:4)	Lipid	Phosphatidylethanolamine (PE)	1.277
gamma-glutamyl-alpha-lysine	Peptide	Gamma-glutamyl Amino Acid	1.274
lactosyl-N-palmitoyl-sphingosine (d18:1/16:0)	Lipid	Lactosylceramides (LCER)	1.270
cystine	Amino Acid	Methionine, Cysteine, SAM and Taurine Metabolism	1.270
4-acetamidobutanoate	Amino Acid	Polyamine Metabolism	1.268
1-carboxyethylphenylalanine	Amino Acid	Phenylalanine Metabolism	1.265
sphingomyelin (d18:2/23:1)	Lipid	Sphingomyelins	1.252
5,6-dihydrouridine	Nucleotide	Pyrimidine Metabolism, Uracil containing	1.251
picolinoylglycine	Lipid	Fatty Acid Metabolism (Acyl Glycine)	1.250
2-hydroxyhippurate (salicylurate)	Xenobiotics	Benzoate Metabolism	1.244
4-hydroxyhippurate	Xenobiotics	Benzoate Metabolism	1.244
androsterone glucuronide	Lipid	Androgenic Steroids	1.243
N6-carbamoylthreonyladenosine	Nucleotide	Purine Metabolism, Adenine containing	1.237
aconitate [cis or trans]	Energy	TCA Cycle	1.235
2-stearoyl-GPE (18:0)	Lipid	Lysophospholipid	1.235
1-stearoyl-2-arachidonoyl-GPC (18:0/20:4)	Lipid	Phosphatidylcholine (PC)	1.234
dimethylglycine	Amino Acid	Glycine, Serine and Threonine Metabolism	1.234
stearoylcarnitine (C18)	Lipid	Fatty Acid Metabolism (Acyl Carnitine, Long Chain Saturated)	1.232
1-methylhistidine	Amino Acid	Histidine Metabolism	1.226
gamma-glutamylphenylalanine	Peptide	Gamma-glutamyl Amino Acid	1.225
3-hydroxyisobutyrate	Amino Acid	Leucine, Isoleucine and Valine Metabolism	1.224
C-glycosyltryptophan	Amino Acid	Tryptophan Metabolism	1.223
5alpha-androstan-3alpha,17beta-diol monosulfate (1)	Lipid	Androgenic Steroids	1.214
pregnanediol-3-glucuronide	Lipid	Progestin Steroids	1.213
5-hydroxyindoleacetate	Amino Acid	Tryptophan Metabolism	1.213
1-linoleoylglycerol (18:2)	Lipid	Monoacylglycerol	1.210
docosadienoate (22:2n6)	Lipid	Long Chain Polyunsaturated Fatty Acid (n3 and n6)	1.210
1,3-dimethylurate	Xenobiotics	Xanthine Metabolism	1.209

BIOCHEMICAL	Super pathway	Sub pathway	M2.VIP[3+2+0]
3-methylglutarylcarnitine (2)	Amino Acid	Leucine, Isoleucine and Valine Metabolism	1.205
dimethylarginine (SDMA + ADMA)	Amino Acid	Urea cycle; Arginine and Proline Metabolism	1.204
3-methylglutaconate	Amino Acid	Leucine, Isoleucine and Valine Metabolism	1.204
3-hydroxy-2-ethylpropionate	Amino Acid	Leucine, Isoleucine and Valine Metabolism	1.204
N1-methyladenosine	Nucleotide	Purine Metabolism, Adenine containing	1.199
1-arachidonylglycerol (20:4)	Lipid	Monoacylglycerol	1.197
proline	Amino Acid	Urea cycle; Arginine and Proline Metabolism	1.193
androsterone sulfate	Lipid	Androgenic Steroids	1.193
sulfate	Xenobiotics	Chemical	1.190
1-linoleoyl-2-linolenoyl-GPC (18:2/18:3)	Lipid	Phosphatidylcholine (PC)	1.186
carnitine	Lipid	Carnitine Metabolism	1.183
alpha-hydroxyisovalerate	Amino Acid	Leucine, Isoleucine and Valine Metabolism	1.183
1-stearoyl-2-docosahexaenoyl-GPC (18:0/22:6)	Lipid	Phosphatidylcholine (PC)	1.181
argininate	Amino Acid	Urea cycle; Arginine and Proline Metabolism	1.179
1-palmitoleoyl-GPC (16:1)	Lipid	Lysophospholipid	1.177
retinol (Vitamin A)	Cofactors and Vitamins	Vitamin A Metabolism	1.176
catechol sulfate	Xenobiotics	Benzoate Metabolism	1.175
phenylalanine	Amino Acid	Phenylalanine Metabolism	1.168
arabonate/xylonate	Carbohydrate	Pentose Metabolism	1.167
1-carboxyethylvaline	Amino Acid	Leucine, Isoleucine and Valine Metabolism	1.167
pipecolate	Amino Acid	Lysine Metabolism	1.167
ascorbic acid 3-sulfate	Cofactors and Vitamins	Ascorbate and Aldarate Metabolism	1.166
sphingomyelin (d18:1/22:2, d18:2/22:1, d16:1/24:2)	Lipid	Sphingomyelins	1.166
2-hydroxy-3-methylvalerate	Amino Acid	Leucine, Isoleucine and Valine Metabolism	1.166
10-nonadecenoate (19:1n9)	Lipid	Long Chain Monounsaturated Fatty Acid	1.165
palmitate (16:0)	Lipid	Long Chain Saturated Fatty Acid	1.164
1-palmitoyl-2-docosahexaenoyl-GPC (16:0/22:6)	Lipid	Phosphatidylcholine (PC)	1.164
threonine	Amino Acid	Glycine, Serine and Threonine Metabolism	1.162
1-myristoyl-2-arachidonoyl-GPC (14:0/20:4)	Lipid	Phosphatidylcholine (PC)	1.159

BIOCHEMICAL	Super pathway	Sub pathway	M2.VIP[3+2+0]
isobutyrylcarnitine (C4)	Amino Acid	Leucine, Isoleucine and Valine Metabolism	1.158
creatinine	Amino Acid	Creatine Metabolism	1.158
oleoylcholine	Lipid	Fatty Acid Metabolism (Acyl Choline)	1.153
1-linolenoyl-GPC (18:3)	Lipid	Lysophospholipid	1.153
10-heptadecenoate (17:1n7)	Lipid	Long Chain Monounsaturated Fatty Acid	1.153
1-palmitoyl-2-linoleoyl-GPC (16:0/18:2)	Lipid	Phosphatidylcholine (PC)	1.153
palmitoylcarnitine (C16)	Lipid	Fatty Acid Metabolism (Acyl Carnitine, Long Chain Saturated)	1.152
myristate (14:0)	Lipid	Long Chain Saturated Fatty Acid	1.151
N-acetylalanine	Amino Acid	Alanine and Aspartate Metabolism	1.151
carotene diol (1)	Cofactors and Vitamins	Vitamin A Metabolism	1.151
dihomo-linoleate (20:2n6)	Lipid	Long Chain Polyunsaturated Fatty Acid (n3 and n6)	1.150
gulonate	Cofactors and Vitamins	Ascorbate and Aldarate Metabolism	1.150
N6-succinyladenosine	Nucleotide	Purine Metabolism, Adenine containing	1.149
gamma-glutamylmethionine	Peptide	Gamma-glutamyl Amino Acid	1.147
3-hydroxybutyroylglycine	Lipid	Fatty Acid Metabolism (Acyl Glycine)	1.147
16a-hydroxy DHEA 3-sulfate	Lipid	Androgenic Steroids	1.147
cysteinylglycine disulfide	Amino Acid	Glutathione Metabolism	1.143
N-acetylserine	Amino Acid	Glycine, Serine and Threonine Metabolism	1.141
1-stearoyl-2-linoleoyl-GPE (18:0/18:2)	Lipid	Phosphatidylethanolamine (PE)	1.141
gamma-glutamylglycine	Peptide	Gamma-glutamyl Amino Acid	1.141
1-palmitoyl-2-palmitoleoyl-GPC (16:0/16:1)	Lipid	Phosphatidylcholine (PC)	1.139
1-methylguanidine	Amino Acid	Guanidino and Acetamido Metabolism	1.139
pentadecanoate (15:0)	Lipid	Long Chain Saturated Fatty Acid	1.134
N-formylmethionine	Amino Acid	Methionine, Cysteine, SAM and Taurine Metabolism	1.133
(14 or 15)-methylpalmitate (a17:0 or i17:0)	Lipid	Fatty Acid, Branched	1.133
eicosenoate (20:1)	Lipid	Long Chain Monounsaturated Fatty Acid	1.132
alpha-ketobutyrate	Amino Acid	Methionine, Cysteine, SAM and Taurine Metabolism	1.132
lysine	Amino Acid	Lysine Metabolism	1.131
5alpha-androstan-3beta,17beta-diol disulfate	Lipid	Androgenic Steroids	1.130
1-methylurate	Xenobiotics	Xanthine Metabolism	1.129

BIOCHEMICAL	Super pathway	Sub pathway	M2.VIP[3+2+0]
5-hydroxylysine	Amino Acid	Lysine Metabolism	1.129
margarate (17:0)	Lipid	Long Chain Saturated Fatty Acid	1.125
1-palmitoyl-GPI (16:0)	Lipid	Lysophospholipid	1.123
carotene diol (3)	Cofactors and Vitamins	Vitamin A Metabolism	1.121
3-methoxytyrosine	Amino Acid	Tyrosine Metabolism	1.120
1-(1-enyl-stearoyl)-2-linoleoyl-GPE (P-18:0/18:2)	Lipid	Plasmalogen	1.119
asparagine	Amino Acid	Alanine and Aspartate Metabolism	1.117
2R,3R-dihydroxybutyrate	Lipid	Fatty Acid, Dihydroxy	1.116
linoleoylcholine	Lipid	Fatty Acid Metabolism (Acyl Choline)	1.115
pyruvate	Carbohydrate	Glycolysis, Gluconeogenesis, and Pyruvate Metabolism	1.114
epiandrosterone sulfate	Lipid	Androgenic Steroids	1.110
methionine	Amino Acid	Methionine, Cysteine, SAM and Taurine Metabolism	1.109
docosapentaenoate (n3 DPA; 22:5n3)	Lipid	Long Chain Polyunsaturated Fatty Acid (n3 and n6)	1.108
1-arachidonoyl-GPC (20:4n6)	Lipid	Lysophospholipid	1.107
1-(1-enyl-palmitoyl)-2-oleoyl-GPC (P-16:0/18:1)	Lipid	Plasmalogen	1.105
urea	Amino Acid	Urea cycle; Arginine and Proline Metabolism	1.105
1-methylxanthine	Xenobiotics	Xanthine Metabolism	1.104
1-stearoyl-2-arachidonoyl-GPI (18:0/20:4)	Lipid	Phosphatidylinositol (PI)	1.101
N,N,N-trimethyl-5-aminovalerate	Amino Acid	Lysine Metabolism	1.101
1-stearoyl-2-linoleoyl-GPC (18:0/18:2)	Lipid	Phosphatidylcholine (PC)	1.100
(S)-a-amino-omega-caprolactam	Xenobiotics	Food Component/Plant	1.099
oleate/vaccenate (18:1)	Lipid	Long Chain Monounsaturated Fatty Acid	1.098
indolelactate	Amino Acid	Tryptophan Metabolism	1.098
1-stearoyl-GPI (18:0)	Lipid	Lysophospholipid	1.096
linoleate (18:2n6)	Lipid	Long Chain Polyunsaturated Fatty Acid (n3 and n6)	1.089
hexadecanedioate (C16-DC)	Lipid	Fatty Acid, Dicarboxylate	1.086
alpha-ketoglutarate	Energy	TCA Cycle	1.086
2-linoleoylglycerol (18:2)	Lipid	Monoacylglycerol	1.085
sphingomyelin (d18:2/24:2)	Lipid	Sphingomyelins	1.083
trans-4-hydroxyproline	Amino Acid	Urea cycle; Arginine and Proline Metabolism	1.082
N6-acetyllysine	Amino Acid	Lysine Metabolism	1.080
carotene diol (2)	Cofactors and Vitamins	Vitamin A Metabolism	1.077
N-acetylputrescine	Amino Acid	Polyamine Metabolism	1.076
stearate (18:0)	Lipid	Long Chain Saturated Fatty Acid	1.076

BIOCHEMICAL	Super pathway	Sub pathway	M2.VIP[3+2+0]
1-linolenoylglycerol (18:3)	Lipid	Monoacylglycerol	1.075
glycerophosphorylcholine (GPC)	Lipid	Phospholipid Metabolism	1.075
prolylglycine	Peptide	Dipeptide	1.074
kynurenine	Amino Acid	Tryptophan Metabolism	1.072
1-(1-enyl-palmitoyl)-2-oleoyl-GPE (P-16:0/18:1)	Lipid	Plasmalogen	1.072
deoxycholic acid 12-sulfate	Lipid	Secondary Bile Acid Metabolism	1.071
9-hydroxystearate	Lipid	Fatty Acid, Monohydroxy	1.071
gamma-glutamylhistidine	Peptide	Gamma-glutamyl Amino Acid	1.067
EDTA	Xenobiotics	Chemical	1.067
4-hydroxyglutamate	Amino Acid	Glutamate Metabolism	1.066
O-sulfo-L-tyrosine	Xenobiotics	Chemical	1.065
succinylcarnitine (C4-DC)	Energy	TCA Cycle	1.064
gamma-glutamyl-2-aminobutyrate	Peptide	Gamma-glutamyl Amino Acid	1.064
1-palmitoleoylglycerol (16:1)	Lipid	Monoacylglycerol	1.064
N-acetyl-beta-alanine	Nucleotide	Pyrimidine Metabolism, Uracil containing	1.062
vanillactate	Amino Acid	Tyrosine Metabolism	1.061
perfluorooctanesulfonate (PFOS)	Xenobiotics	Chemical	1.061
pentose acid	Partially Characterized Molecules	Partially Characterized Molecules	1.061
1-arachidonoyl-GPI (20:4)	Lipid	Lysophospholipid	1.060
tetradecadienedioate (C14:2-DC)	Lipid	Fatty Acid, Dicarboxylate	1.060
17alpha-hydroxypregnenolone 3-sulfate	Lipid	Pregnenolone Steroids	1.058
palmitoleate (16:1n7)	Lipid	Long Chain Monounsaturated Fatty Acid	1.058
1-palmitoyl-2-arachidonoyl-GPI (16:0/20:4)	Lipid	Phosphatidylinositol (PI)	1.057
arachidonate (20:4n6)	Lipid	Long Chain Polyunsaturated Fatty Acid (n3 and n6)	1.056
1-stearoyl-2-linoleoyl-GPI (18:0/18:2)	Lipid	Phosphatidylinositol (PI)	1.056
azelate (C9-DC)	Lipid	Fatty Acid, Dicarboxylate	1.053
serine	Amino Acid	Glycine, Serine and Threonine Metabolism	1.052
glucuronide of piperine metabolite C17H21NO3 (4)	Xenobiotics	Food Component/Plant	1.051
eicosapentaenoate (EPA; 20:5n3)	Lipid	Long Chain Polyunsaturated Fatty Acid (n3 and n6)	1.051
1-(1-enyl-palmitoyl)-2-arachidonoyl-GPE (P-16:0/20:4)	Lipid	Plasmalogen	1.050
3-(4-hydroxyphenyl)lactate	Amino Acid	Tyrosine Metabolism	1.050
homostachydrine	Xenobiotics	Food Component/Plant	1.047
3-hydroxyhexanoate	Lipid	Fatty Acid, Monohydroxy	1.045
deoxycarnitine	Lipid	Carnitine Metabolism	1.045

BIOCHEMICAL	Super pathway	Sub pathway	M2.VIP[3+2+0]
alanine	Amino Acid	Alanine and Aspartate Metabolism	1.044
N-acetyltryptophan	Amino Acid	Tryptophan Metabolism	1.043
11beta-hydroxyandrosterone glucuronide	Lipid	Androgenic Steroids	1.036
3-(3-amino-3-carboxypropyl)uridine	Nucleotide	Pyrimidine Metabolism, Uracil containing	1.036
ribitol	Carbohydrate	Pentose Metabolism	1.036
2-palmitoyl-GPC (16:0)	Lipid	Lysophospholipid	1.035
1-linoleoyl-GPI (18:2)	Lipid	Lysophospholipid	1.035
octadecenedioate (C18:1-DC)	Lipid	Fatty Acid, Dicarboxylate	1.034
2-methylbutyrylcarnitine (C5)	Amino Acid	Leucine, Isoleucine and Valine Metabolism	1.034
stearidonate (18:4n3)	Lipid	Long Chain Polyunsaturated Fatty Acid (n3 and n6)	1.034
3-hydroxybutyrate (BHBA)	Lipid	Ketone Bodies	1.034
docosapentaenoate (n6 DPA; 22:5n6)	Lipid	Long Chain Polyunsaturated Fatty Acid (n3 and n6)	1.033
(16 or 17)-methylstearate (a19:0 or i19:0)	Lipid	Fatty Acid, Branched	1.031
docosahexaenoate (DHA; 22:6n3)	Lipid	Long Chain Polyunsaturated Fatty Acid (n3 and n6)	1.030
N-acetyltaurine	Amino Acid	Methionine, Cysteine, SAM and Taurine Metabolism	1.029
1-oleoyl-GPI (18:1)	Lipid	Lysophospholipid	1.022
linolenate [alpha or gamma; (18:3n3 or 6)]	Lipid	Long Chain Polyunsaturated Fatty Acid (n3 and n6)	1.018
N-acetyltheanine	Xenobiotics	Food Component/Plant	1.018
palmitoyl ethanolamide	Lipid	Endocannabinoid	1.016
branched-chain, straight-chain, or cyclopropyl 10:1 fatty acid (1)	Partially Characterized Molecules	Partially Characterized Molecules	1.016
7-methylxanthine	Xenobiotics	Xanthine Metabolism	1.016
N-acetylcarnosine	Amino Acid	Histidine Metabolism	1.015
gamma-glutamylglutamine	Peptide	Gamma-glutamyl Amino Acid	1.013
5alpha-androstan-3alpha,17beta-diol disulfate	Lipid	Androgenic Steroids	1.012
sphinganine-1-phosphate	Lipid	Sphingolipid Synthesis	1.012
eicosenedioate (C20:1-DC)	Lipid	Fatty Acid, Dicarboxylate	1.011
ornithine	Amino Acid	Urea cycle; Arginine and Proline Metabolism	1.010
oleoyl ethanolamide	Lipid	Endocannabinoid	1.010
S-adenosylhomocysteine (SAH)	Amino Acid	Methionine, Cysteine, SAM and Taurine Metabolism	1.010
pyroglutamine	Amino Acid	Glutamate Metabolism	1.010
1-myristoyl-2-palmitoyl-GPC (14:0/16:0)	Lipid	Phosphatidylcholine (PC)	1.009
cystathionine	Amino Acid	Methionine, Cysteine, SAM and Taurine Metabolism	1.008

BIOCHEMICAL	Super pathway	Sub pathway	M2.VIP[3+2+0]
gamma-glutamylglutamate	Peptide	Gamma-glutamyl Amino Acid	1.008
1-palmitoyl-2-linoleoyl-GPI (16:0/18:2)	Lipid	Phosphatidylinositol (PI)	1.007
imidazole propionate	Amino Acid	Histidine Metabolism	1.005
glutamine	Amino Acid	Glutamate Metabolism	1.003
sphingosine 1-phosphate	Lipid	Sphingosines	1.003
salicyluric glucuronide	Xenobiotics	Drug - Analgesics, Anesthetics	1.003
3,4-dihydroxybutyrate	Lipid	Fatty Acid, Dihydroxy	1.002
caprate (10:0)	Lipid	Medium Chain Fatty Acid	1.001
N-acetyl-1-methylhistidine	Amino Acid	Histidine Metabolism	1.001
oleoylcarnitine (C18:1)	Lipid	Fatty Acid Metabolism (Acyl Carnitine, Monounsaturated)	1.000
