# Supplementary material for: Metabolomics Profiling of Stages of Coronary Artery Disease Progression
Source: Metabolites. 2024 May 22;14(6):292. doi: 10.3390/metabo14060292 (PMC11205943; doi:10.3390/metabo14060292)
Supplement: Supplementary file 1 [file metabolites-14-00292-s001.zip › Table S4- Linear regression results.pdf]

Table S4. Linear regression analysis. The table shows nominally significant metabolites of linear regression analysis. p<0.05 considered as significant.

**Metabolites**	**Subpathway**	**Superpathway**	**Estimate**	**Standard Error**	***p*-value**	**FDR**
mannitol/sorbitol	Fructose, Mannose and Galactose Metabolism	Carbohydrate	0.4473	0.0965	<0.0001	0.0053
glucose	Glycolysis, Gluconeogenesis, and Pyruvate Metabolism	Carbohydrate	0.1555	0.0409	0.0002	0.0622
pregnenediol sulfate (C21H34O5S)	Pregnenolone Steroids	Lipid	-0.3141	0.0835	0.0002	0.0622
mannose	Fructose, Mannose and Galactose Metabolism	Carbohydrate	0.1710	0.0477	0.0004	0.0784
oleoylcarnitine (C18:1)	Fatty Acid Metabolism (Acyl Carnitine, Monounsaturated)	Lipid	-0.1496	0.0420	0.0005	0.0784
ribitol	Pentose Metabolism	Carbohydrate	0.1365	0.0395	0.0007	0.0924
quinolinate	Nicotinate and Nicotinamide Metabolism	Cofactors and Vitamins	-0.1502	0.0440	0.0008	0.0924
pregnenediol disulfate (C21H34O8S2)	Pregnenolone Steroids	Lipid	-0.2826	0.0874	0.0014	0.1333
21-hydroxypregnenolone disulfate	Pregnenolone Steroids	Lipid	-0.2401	0.0743	0.0014	0.1333
pregnenetriol sulfate	Pregnenolone Steroids	Lipid	-0.2443	0.0763	0.0016	0.1333
deoxycarnitine	Carnitine Metabolism	Lipid	-0.0815	0.0263	0.0022	0.1692
androstenediol (3alpha, 17alpha) monosulfate (2)	Androgenic Steroids	Lipid	-0.2350	0.0768	0.0025	0.1774
dehydroepiandrosterone sulfate (DHEA-S)	Androgenic Steroids	Lipid	-0.2880	0.0952	0.0028	0.1832
1,5-anhydroglucitol (1,5-AG)	Glycolysis, Gluconeogenesis, and Pyruvate Metabolism	Carbohydrate	-0.3712	0.1252	0.0034	0.1970
lactosyl-N-palmitoyl-sphingosine (d18:1/16:0)	Lactosylceramides (LCER)	Lipid	-0.0855	0.0291	0.0036	0.1970
1-palmitoyl-2-docosahexaenoyl-GPE (16:0/22:6)	Phosphatidylethanolamine (PE)	Lipid	0.1725	0.0588	0.0037	0.1970
linoleoylcarnitine (C18:2)	Fatty Acid Metabolism (Acyl Carnitine, Polyunsaturated)	Lipid	-0.1265	0.0434	0.0039	0.1970

16a-hydroxy DHEA 3-sulfate	Androgenic Steroids	Lipid	-0.3082	0.1065	0.0042	0.1994
6-bromotryptophan	Tryptophan Metabolism	Amino Acid	-0.1035	0.0367	0.0052	0.2161
glucuronate	Aminosugar Metabolism	Carbohydrate	0.1252	0.0444	0.0053	0.2161
N-stearoyl-sphingosine (d18:1/18:0)	Ceramides	Lipid	0.1143	0.0406	0.0053	0.2161
androstenediol (3beta,17beta) disulfate (2)	Androgenic Steroids	Lipid	-0.1854	0.0665	0.0058	0.2234
kynurenine	Tryptophan Metabolism	Amino Acid	-0.0855	0.0311	0.0065	0.2248
1-stearoyl-2-arachidonoyl-GPE (18:0/20:4)	Phosphatidylethanolamine (PE)	Lipid	0.1276	0.0465	0.0066	0.2248
palmitoylcarnitine (C16)	Fatty Acid Metabolism (Acyl Carnitine, Long Chain Saturated)	Lipid	-0.0962	0.0351	0.0066	0.2248
mannonate	Food Component/Plant	Xenobiotics	0.1228	0.0458	0.0079	0.2571
eicosanedioate (C20-DC)	Fatty Acid, Dicarboxylate	Lipid	-0.1323	0.0497	0.0084	0.2578
erythritol	Food Component/Plant	Xenobiotics	0.1055	0.0397	0.0085	0.2578
eicosenedioate (C20:1-DC)	Fatty Acid, Dicarboxylate	Lipid	-0.2002	0.0773	0.0103	0.3024
p-cresol glucuronide	Tyrosine Metabolism	Amino Acid	0.3534	0.1383	0.0114	0.3229
branched-chain, straight-chain, or cyclopropyl 10:1 fatty acid (1)	Partially Characterized Molecules	Partially Characterized Molecules	-0.1888	0.0757	0.0134	0.3355
1-palmitoyl-2-arachidonoyl-GPE (16:0/20:4)	Phosphatidylethanolamine (PE)	Lipid	0.1115	0.0448	0.0136	0.3355
glycosyl ceramide (d18:1/20:0, d16:1/22:0)	Hexosylceramides (HCER)	Lipid	-0.1076	0.0433	0.0139	0.3355
3-formylindole	Food Component/Plant	Xenobiotics	-0.0777	0.0314	0.0140	0.3355
N,N,N-trimethyl-alanylproline betaine (TMAP)	Urea cycle; Arginine and Proline Metabolism	Amino Acid	-0.0673	0.0272	0.0141	0.3355
metformin	Drug - Metabolic	Xenobiotics	1.2427	0.4989	0.0152	0.3355
stearoylcarnitine (C18)	Fatty Acid Metabolism (Acyl Carnitine, Long Chain Saturated)	Lipid	-0.0933	0.0382	0.0154	0.3355
retinol (Vitamin A)	Vitamin A Metabolism	Cofactors and Vitamins	0.0754	0.0310	0.0158	0.3355
2-stearoyl-GPE (18:0)	Lysophospholipid	Lipid	0.1299	0.0534	0.0158	0.3355

gluconate	Food Component/Plant	Xenobiotics	0.1133	0.0467	0.0161	0.3355
2'-deoxyuridine	Pyrimidine Metabolism, Uracil containing	Nucleotide	-0.1375	0.0565	0.0162	0.3355
pregnenetriol disulfate	Pregnenolone Steroids	Lipid	-0.2000	0.0832	0.0171	0.3379
gamma-glutamylcitrulline	Gamma-glutamyl Amino Acid	Peptide	-0.1162	0.0484	0.0171	0.3379
erythronate	Aminosugar Metabolism	Carbohydrate	0.0585	0.0245	0.0179	0.3394
pantoprazole	Drug - Gastrointestinal	Xenobiotics	4.4875	0.6126	0.0181	0.3394
GlcNAc sulfate conjugate of C21H34O2 steroid	Partially Characterized Molecules	Partially Characterized Molecules	-0.2334	0.0979	0.0184	0.3394
glycosyl-N-palmitoyl-sphingosine (d18:1/16:0)	Hexosylceramides (HCER)	Lipid	-0.0731	0.0309	0.0190	0.3445
2'-O-methylcytidine	Pyrimidine Metabolism, Cytidine containing	Nucleotide	-0.0955	0.0407	0.0198	0.3502
3-hydroxyhexanoate	Fatty Acid, Monohydroxy	Lipid	-0.0996	0.0426	0.0202	0.3502
kynurenate	Tryptophan Metabolism	Amino Acid	-0.0891	0.0385	0.0217	0.3695
androstenediol (3beta,17beta) monosulfate (1)	Androgenic Steroids	Lipid	-0.1922	0.0835	0.0224	0.3732
1-palmitoyl-2-oleoyl-GPE (16:0/18:1)	Phosphatidylethanolamine (PE)	Lipid	0.1310	0.0573	0.0233	0.3811
2R,3R-dihydroxybutyrate	Fatty Acid, Dihydroxy	Lipid	0.0955	0.0421	0.0242	0.3880
3-methoxytyrosine	Tyrosine Metabolism	Amino Acid	-0.0766	0.0340	0.0250	0.3937
5alpha-pregnan-3beta,20beta-diol monosulfate (1)	Progestin Steroids	Lipid	-0.2408	0.1072	0.0257	0.3979
hexadecenedioate (C16:1-DC)	Fatty Acid, Dicarboxylate	Lipid	-0.1273	0.0570	0.0267	0.4054
fructose	Fructose, Mannose and Galactose Metabolism	Carbohydrate	0.0962	0.0436	0.0285	0.4160
sphingomyelin (d18:1/18:1, d18:2/18:0)	Sphingomyelins	Lipid	0.0485	0.0221	0.0291	0.4160
4-methylguaiacol sulfate	Benzoate Metabolism	Xenobiotics	0.3349	0.1517	0.0292	0.4160
2-methoxyhydroquinone sulfate (2)	Benzoate Metabolism	Xenobiotics	-0.2392	0.1058	0.0294	0.4160
guaiacol sulfate	Benzoate Metabolism	Xenobiotics	0.1406	0.0644	0.0301	0.4190
glycerophosphoethanolamine	Phospholipid Metabolism	Lipid	0.0518	0.0238	0.0310	0.4198
1-methyl-4-imidazoleacetate	Histidine Metabolism	Amino Acid	0.0714	0.0329	0.0311	0.4198

cytosine	Pyrimidine Metabolism, Cytidine containing	Nucleotide	0.2412	0.1110	0.0318	0.4219
dimethyl sulfone	Chemical	Xenobiotics	-0.2359	0.1094	0.0323	0.4219
(N(1) + N(8))-acetylspermidine	Polyamine Metabolism	Amino Acid	-0.0787	0.0368	0.0339	0.4298
1-carboxyethylphenylalanine	Phenylalanine Metabolism	Amino Acid	0.1066	0.0502	0.0349	0.4298
pregnenolone sulfate	Pregnenolone Steroids	Lipid	-0.1930	0.0910	0.0351	0.4298
sphingomyelin (d18:2/23:1)	Sphingomyelins	Lipid	-0.0669	0.0318	0.0362	0.4298
gamma-CEHC	Tocopherol Metabolism	Cofactors and Vitamins	0.1316	0.0626	0.0369	0.4298
sphingomyelin (d18:2/18:1)	Sphingomyelins	Lipid	-0.0601	0.0287	0.0376	0.4298
daidzein sulfate (2)	Food Component/Plant	Xenobiotics	-2.1896	0.4382	0.0378	0.4298
caprylate (8:0)	Medium Chain Fatty Acid	Lipid	-0.1212	0.0580	0.0379	0.4298
2-hydroxyhippurate (salicylurate)	Benzoate Metabolism	Xenobiotics	0.3413	0.1637	0.0384	0.4298
5-dodecenoylcarnitine (C12:1)	Fatty Acid Metabolism (Acyl Carnitine, Monounsaturated)	Lipid	-0.1143	0.0550	0.0389	0.4298
stearoyl sphingomyelin (d18:1/18:0)	Sphingomyelins	Lipid	0.0467	0.0225	0.0391	0.4298
3,4-dihydroxybutyrate	Fatty Acid, Dihydroxy	Lipid	0.0867	0.0418	0.0392	0.4298
1-palmitoyl-GPE (16:0)	Lysophospholipid	Lipid	0.0743	0.0359	0.0394	0.4298
N-acetylleucine	Leucine, Isoleucine and Valine Metabolism	Amino Acid	-0.0573	0.0277	0.0400	0.4305
naproxen	Drug - Analgesics, Anesthetics	Xenobiotics	15.5252	1.0328	0.0423	0.4451
andro steroid monosulfate C19H28O6S (1)	Androgenic Steroids	Lipid	-0.2140	0.1048	0.0424	0.4451
myristoleate (14:1n5)	Long Chain Monounsaturated Fatty Acid	Lipid	-0.1491	0.0732	0.0429	0.4451
N,N,N-trimethyl-5-aminovalerate	Lysine Metabolism	Amino Acid	0.0894	0.0442	0.0443	0.4490
palmitoleoylcarnitine (C16:1)	Fatty Acid Metabolism (Acyl Carnitine, Monounsaturated)	Lipid	-0.1136	0.0562	0.0450	0.4490
sphingomyelin (d18:2/24:1, d18:1/24:2)	Sphingomyelins	Lipid	-0.0534	0.0265	0.0450	0.4490
1-stearoyl-GPE (18:0)	Lysophospholipid	Lipid	0.0725	0.0362	0.0463	0.4490

citrulline	Urea cycle; Arginine and Proline Metabolism	Amino Acid	-0.0521	0.0260	0.0469	0.4490
metabolonic lactone sulfate	Partially Characterized Molecules	Partially Characterized Molecules	-0.1761	0.0881	0.0469	0.4490
gulonate	Ascorbate and Aldarate Metabolism	Cofactors and Vitamins	0.1057	0.0530	0.0474	0.4490
2-O-methylascorbic acid	Ascorbate and Aldarate Metabolism	Cofactors and Vitamins	0.0767	0.0386	0.0480	0.4490
pipecolate	Lysine Metabolism	Amino Acid	0.1046	0.0526	0.0481	0.4490
indoleacetate	Tryptophan Metabolism	Amino Acid	-0.1220	0.0616	0.0491	0.4534
8-methoxykynurenate	Tryptophan Metabolism	Amino Acid	0.1525	0.0767	0.0498	0.4548
