# Supplementary material for: Metabolomics Profiling of Stages of Coronary Artery Disease Progression
Source: Metabolites. 2024 May 22;14(6):292. doi: 10.3390/metabo14060292 (PMC11205943; doi:10.3390/metabo14060292)
Supplement: Supplementary file 1 [file metabolites-14-00292-s001.zip › Table S5-Linear regression analysis without correcting for age -NRA.pdf]

Table S5: Linear regression analysis without correcting for age. Table shows the metabolites that are associated with different stages of disease.

metabolites	Sub-pathway	Super-pathway	Estimate	SE	p-value	FDR
pregnenediol sulfate (C21H34O5S)	Pregnenolone Steroids	Lipid	-0.389	0.073	<0.001	<0.001
mannitol/sorbitol	Fructose, Mannose and Galactose Metabolism	Carbohydrate	0.350	0.085	<0.001	0.004
oleoylcarnitine (C18:1)	Fatty Acid Metabolism (Acyl Carnitine, Monounsaturated)	Lipid	-0.145	0.036	<0.001	0.004
mannose	Fructose, Mannose and Galactose Metabolism	Carbohydrate	0.165	0.042	<0.001	0.005
glucose	Glycolysis, Gluconeogenesis, and Pyruvate Metabolism	Carbohydrate	0.141	0.036	<0.001	0.005
ribitol	Pentose Metabolism	Carbohydrate	0.104	0.034	0.003	0.044
quinolinate	Nicotinate and Nicotinamide Metabolism	Cofactors and Vitamins	-0.073	0.039	0.065	0.340
