# Supplementary material for: Metabolomics Profiling of Stages of Coronary Artery Disease Progression
Source: Metabolites. 2024 May 22;14(6):292. doi: 10.3390/metabo14060292 (PMC11205943; doi:10.3390/metabo14060292)
Supplement: Supplementary file 1 [file metabolites-14-00292-s001.zip › Table S6- List of medications used-GA.pdf]

Table S6. List of medications used by the disease groups. The table includes a checklist of antidiabetic, antihypertensive and cholesterol lowering medication use. Some medications were labelled as the patients reported. Empty rows indicate absence of information.

**Group ID**	**Medication name 1**	**Medication name 2**	**Medication name 3**	**Medication name 4**	**Medication name 5**	**Medication name 6**	**Medication name 7**	**Medication name 8**
Coronary artery disease 1	Aspirin	metformin	dipeptidyl peptidase-4 inhibitor (antidiabetic)	Insulin injection	sulfonylurea	sulfonylurea	statin	Pregabalin
Coronary artery disease 2	Sulfonylurea	thiazolidinedione (antidiabetic)	metformin	statin	angiotensin II receptor blocker, thiazide diuretic	Beta-blocker	Aspirin	bydureon (antidiabetic)
Coronary artery disease 3	angiotensin II receptor blocker, thiazide diuretic	statin	statin	Pregabalin	diclofenac potassium (NSAID)	Proton Pump Inhibitors	calcium channel blocker, thiazide diuretic	
Coronary artery disease 4	PLATELET AGGREGATION INHIBITOR	Aspirin 100mg	Beta Blocker, thiazide diuretic	statin 20mg OD	ANTIDIABETIC MEDICATION	ACE inhibitor	ISDN (Isosorbidedinitrate-vasodilator for angina)	
Coronary artery disease 5	PLATELET AGGREGATION INHIBITOR	Proton Pump Inhibitors	metformin	statin	aspirin	Beta Blocker, thiazide diuretic	Eye Drops for Moisturization	
Coronary artery disease 6	Insulin injection	metformin Tablet	ANTIHYPERTENSIVE MEDICATION	statin	vitamin d tablet	Omega 3 tablet	Aspirin	
Coronary artery disease 7	asprin	Insulin injection	metformin	Heart condition tab.	ANTIHYPERTENSIVE MEDICATION	ranitidine, histamine-2 blocker		

Coronary artery disease 8	ACE inhibitor	CHOLESTEROL LOWERING MEDICATION	aspirin	Beta-blocker	omnex for prostatic enlargement	Avodart tab.		
Coronary artery disease 9	CHOLESTEROL LOWERING MEDICATION	Insulin	ANTIDIABETIC MEDICATION	thyroid hormone replacement	Vit D tab weekly			
Coronary artery disease 10	Genovia 100mg once daily	Aspirin 100mg once daily	PLATELET AGGREGATION INHIBITOR	Beta Blocker, thiazide diuretic	omega-3			
Coronary artery disease 11	thyroid hormone replacement	CHOLESTEROL LOWERING MEDICATION	Heart medicine	colon Medicine	Knee Medicine			
Coronary artery disease 12	aspirin	PLATELET AGGREGATION INHIBITOR	Beta Blocker, thiazide diuretic	statin	ANTIDIABETIC MEDICATION			
Coronary artery disease 13	Beta Blocker	Alpha Blocker	Statin	omega 3	PLATELET AGGREGATION INHIBITOR			
Coronary artery disease 14	metformin 1000mg	Sulfonylurea	Aspirin	Beta Blocker, thiazide diuretic	anticoagulant agent unknown for pp			
Coronary artery disease 15	Beta Blocker	asprin	statin	vit D	ACE inhibitor			

Coronary artery disease 16	Metformin 1000mgtab. twice daily	dipeptidyl peptidase-4 inhibitor (antidiabetic)	calcium channel blocker	CHOLESTEROL LOWERING MEDICATION				
Coronary artery disease 17	Insulin Inj	ANTIDIABETI C MEDICATION	ANTIHYPERTE NSIVE MEDICATION	aspirin				
Coronary artery disease 18	asprin	metformin	sulfonylurea	ANTIHYPERTE NSIVE MEDICATION				
Coronary artery disease 19	CHOLESTEROL LOWERING MEDICATION	thyroid hormone replacement	vit D weekly					
Coronary artery disease 20	CHOLESTEROL LOWERING MEDICATION	Insulin	unknown medication for heart					
Coronary artery disease 21	calcium channel blocker	statin	ACE inhibitor					
Coronary artery disease 22								
Coronary artery disease 23								
Coronary artery disease 24	ANTIHYPERTE NSIVE MEDICATION	Heart medicine						

Coronary artery disease 25	aspirin							
Coronary artery disease 26	asprin tab	PLATELET AGGREGATIO N INHIBITOR						
Coronary artery disease 27	Beta Blocker, thiazide diuretic	Folic Acid						
Coronary artery disease 28	CHOLESTEROL LOWERING MEDICATION							
Coronary artery disease 29	calcium channel blocker							
Coronary artery disease 30	Prednisolone	calcium channel blocker, angiotensin II receptor blocker						
Coronary artery disease 31	statin	calcium channel blocker						
Coronary artery disease 32	statin	aspirin						
High Cholesterol 1	Vitamin D tab.	Vitamin fo skin	Vitamin for hair loss					
High Cholesterol 2	Vitamin C	Vitamin D	Folic Acid					

High Cholesterol 3	angiotensin II receptor blocker	Aspirin	CHOLESTEROL LOWERING MEDICATION					
High Cholesterol 4	omega 3	vit D	Calcium tab.					
High Cholesterol 5								
High Cholesterol 6								
High Cholesterol 7								
High Cholesterol8								
High Cholesterol 9								
High Cholesterol 10								
High Cholesterol 11								
High Cholesterol 12								
High Cholesterol 13								
High Cholesterol 14								
High Cholesterol 15								
High Cholesterol 16								
High Cholesterol 17								
High Cholesterol 18								
High Cholesterol 19	Calcium and Vitamin D							

High Cholesterol 20	Contraceptive pill.							
High Cholesterol 21	eye drop							
High Cholesterol 22	Inhalers							
High Cholesterol 23	Iron infusion by injection every 5 days							
High Cholesterol 24	Multivitamin tab	omega 3						
High Cholesterol 25	Multivitamins							
High Cholesterol 26	Multivitamins tab.	Omega 3 tab						
High Cholesterol 27	Nasal Spray							
High Cholesterol 28	ranitidine, histamine-2 blocker	Antibiotics						
High Cholesterol 29	statin 10mg once daily							
High Cholesterol 30	thyroid hormone replacement							
High Cholesterol 31	Vit D							
High Cholesterol 32	vit D	Metformin						
High Cholesterol 33	vit D							
High Cholesterol 34	Vit D tab.							
High Cholesterol 35	Vit.D							

High Cholesterol + complications 1	Insulin NovoMix 30	Beta-blocker	angiotensin II receptor blocker	Tricyclic antidepressant	ANTICONVU LSANT	PLATELET AGGREGATION INHIBITOR	Vitamin B 1+6+12	metformin
High Cholesterol + complications 2	statin	repaglinide (antidiabetic)	dipeptidyl peptidase-4 inhibitor (antidiabetic)	Vit B complex	Ferrous Sulphate	metformin	Insulin Inj	Lipanthyl tablet (weight reduction)
High Cholesterol + complications 3	Insulin injection	metformin	asprin	omega 3	Proton Pump Inhibitors	ANTIHYPERTE NSIVE MEDICATION	Calcium	CHOLESTEROL LOWERING MEDICATION
High Cholesterol + complications 4	angiotensin receptor blocker	thyroid hormone replacement	Vitamin D	Vitamin C	vitamin for hair and nails	Biotin tab.	vitamin for bone	Calcium tab
High Cholesterol + complications 5	Metformin, dipeptidyl peptidase-4 inhibitor (antidiabetic)	Sulfonylurea	Proton Pump Inhibitors	angiotensin II receptor blocker, thiazide diuretic	statin tablet	angiotensin receptor blocker, calcium channel blocker	Vitamin B complex	Aspirin
High Cholesterol + complications 6	Beta Blocker, thiazide diuretic	sulfonylurea	Aspirin 100mg OD	Metformin, dipeptidyl peptidase-4 inhibitor (antidiabetic)	PLATELET AGGREGATI ON INHIBITOR	angiotensin II receptor blocker	statin 20mg OD	
High Cholesterol + complications 7	interferon injection	Vitamin E cap.	Iron tab.	Calcium	Vitamin B12	Spasmocanulase (Methixene Hydrochloride)	ranitidine, histamine-2 blocker	

High Cholesterol + complications 8	ANTIDIABETIC MEDICATION	statin tab.	metformin tab.	Calcium tab.	Vitamin B tab.	Vitamin D tab.		
High Cholesterol + complications 9	Insulin Novomix	metformin	statin	angiotensin receptor blocker	Aspirin 75	VIT B		
High Cholesterol + complications 10	metformin	Sulfonylurea	Vitamin D tab	Calcium tab.	Vitamin C tab	CHOLESTEROL LOWERING MEDICATION		
High Cholesterol + complications 11	Vitamion D	Calcium tablet	ANTIDIABETIC MEDICATION	CHOLESTEROL LOWERING MEDICATION	ANTIHYPERT ENSIVE MEDICATION			
High Cholesterol + complications 12	Insulin injection	metformin Tablet	iron tablet	Vitamind D tab.				
High Cholesterol + complications 13	Beta Blocker, thiazide diuretic	Genomin tablet	CHOLESTEROL LOWERING MEDICATION	vitamin d tablet				
High Cholesterol + complications 14	ANTIHYPERTE NSIVE MEDICATION	metformin	CHOLESTEROL LOWERING MEDICATION	Vitamin D tab.				
High Cholesterol + complications 15	CHOLESTEROL LOWERING MEDICATION	Metformin 500mg tab.	dipeptidyl peptidase-4 inhibitor (antidiabetic)	thyroid hormone replacement				

High Cholesterol + complications 16	ANTIDIABETIC MEDICATION	thyroid hormone replacement	vit D	Paracetamol				
High Cholesterol + complications 17	sulfonylurea	Metformin	GENOVIA	Multi Vitamins				
High Cholesterol + complications 18	CHOLESTEROL LOWERING MEDICATION	Vitamin B12 tab.	Aspirin	Iron tab.				
High Cholesterol + complications 19	vit D	calicum tab	Multivitamin tab	Folic Acid				
High Cholesterol + complications 20	ANTIDIABETIC MEDICATION	insuli injection	statin	ANTIHYPERTENSIVE MEDICATION				
High Cholesterol + complications 21	metformin tab.	statin	zorot					
High Cholesterol + complications 22	ANTIDIABETIC MEDICATION	Multivitamins tab.	Vitamin D tab.					
High Cholesterol + complications 23	Multivitamins tab.	Vitamin B complex	Vitamin D tab.					
High Cholesterol + complications 24	ANTIHYPERTENSIVE MEDICATION	ANTIHYPERTENSIVE MEDICATION	vit D					
High Cholesterol + complications 25	Insulin injection	CHOLESTEROL LOWERING MEDICATION	Ventolin inhaler					

High Cholesterol + complications 26	Metformin	ANTIDIABETI C MEDICATION	thyroid hormone replacement					
High Cholesterol + complications 27	Viamin D	angiotensin II receptor blocker, thiazide diuretic	thyroid hormone replacement					
High Cholesterol + complications 28	Vit D once weekly	Proton Pump Inhibitors	ranitidine, histamine-2 blocker 50 mg Tab					
High Cholesterol + complications 29	asprin tab	metformin	multi vitamin					
High Cholesterol + complications 30	thyroid hormone replacement	thyroid hormone replacement	metformin 500mg tab					
High Cholesterol + complications 31	Sulfonylurea tab	metformin	Genovia tablet					
High Cholesterol + complications 32	metformin tab	vit D tab	Ferrous Sulfate					
High Cholesterol + complications 33	angiotensin II receptor blocker	metformin	CHOLESTEROL LOWERING MEDICATION					
High Cholesterol + complications 34	thyroid hormone replacement	antihypertensiv e	Celecoxib (NSAID)					
High Cholesterol + complications 35	ANTIHYPERLI PEDEMIA	ANTIDIABETI C MEDICATION	asprin tab					

High Cholesterol + complications 36	Multivitamins tab.	Vitamin C tab.	Aspirin					
High Cholesterol + complications 37	ANTIHYPERTE NSIVE MEDICATION	CHOLESTERO L LOWERING MEDICATION	Aspirin					
High Cholesterol + complications 38								
High Cholesterol + complications 39								
High Cholesterol + complications 40								
High Cholesterol + complications 41								
High Cholesterol + complications 42								
High Cholesterol + complications 43								
High Cholesterol + complications 44								
High Cholesterol + complications 45								
High Cholesterol + complications 46								
High Cholesterol + complications 47								
High Cholesterol + complications 48								
High Cholesterol + complications 49								
High Cholesterol + complications 50								
High Cholesterol + complications 51								

High Cholesterol + complications 52								
High Cholesterol + complications 53								
High Cholesterol + complications 54								
High Cholesterol + complications 55								
High Cholesterol + complications 56								
High Cholesterol + complications 57								
High Cholesterol + complications 58								
High Cholesterol + complications 59								
High Cholesterol + complications 60								
High Cholesterol + complications 61								
High Cholesterol + complications 62								
High Cholesterol + complications 63								
High Cholesterol + complications 64								
High Cholesterol + complications 65								
High Cholesterol + complications 66								
High Cholesterol + complications 67								
High Cholesterol + complications 68								

High Cholesterol + complications 69								
High Cholesterol + complications 70								
High Cholesterol + complications 71								
High Cholesterol + complications 72								
High Cholesterol + complications 73								
High Cholesterol + complications 74								
High Cholesterol + complications 75								
High Cholesterol + complications 76	angiotensin II receptor blocker, calcium channel blocker							
High Cholesterol + complications 77	ANTIHYPERTENSIVE MEDICATION							
High Cholesterol + complications 78	ANTIHYPERTENSIVE MEDICATION	Tablets for injection						
High Cholesterol + complications 79	ANTIHYPERTENSIVE MEDICATION							

High Cholesterol + complications 80	ANTIVIRAL MEDICATION	ANTIHYPERTENSIVE MEDICATION							
High Cholesterol + complications 81	Aspirin 50mg daily								
High Cholesterol + complications 82	asprin tab								
High Cholesterol + complications 83	Asprin100	ACE inhibitor							
High Cholesterol + complications 84	CHOLESTEROL LOWERING MEDICATION	Metformin							
High Cholesterol + complications 85	angiotensin II receptor blocker, thiazide diuretic	Zyrtec							
High Cholesterol + complications 86	collagen for joint pain								
High Cholesterol + complications 87	Metformin	statin (Atorvastatin)							
High Cholesterol + complications 88	ANTIDIABETIC MEDICATION								
High Cholesterol + complications 89	ANTIDIABETIC MEDICATION	aspirin							

High Cholesterol + complications 90	ANTIHYPERTENSIVE MEDICATION	ACE inhibitor						
High Cholesterol + complications 91	Insulin	Insulin						
High Cholesterol + complications 92	Insulin injection							
High Cholesterol + complications 93	proton pump inhibitor							
High Cholesterol + complications 94	ANTIHYPERTENSIVE MEDICATION							
High Cholesterol + complications 95	metformin							
High Cholesterol + complications 96	metformin	statin						
High Cholesterol + complications 97	Metformin							
High Cholesterol + complications 98	Metformin	Sulfonylurea						
High Cholesterol + complications 99	Metformin 500mg							
High Cholesterol + complications 100	metformin tab.							
High Cholesterol + complications 101	metformin tab.500mg trice daily	Vitamin B once						
High Cholesterol + complications 102	Multivitamins tab.							
High Cholesterol + complications 103	Multivitamins tablets	Vitamins for hair						

High Cholesterol + complications 104	PANADOL	multivitamin						
High Cholesterol + complications 105	SSRI antidepressant	Multivimans, daily						
High Cholesterol + complications 106	statin 10 mg	Aspirin 100mg						
High Cholesterol + complications 107	statin 10 mg							
High Cholesterol + complications	statin tab.	Vitamin D						
High Cholesterol + complications 108	thyroid hormone replacement							
High Cholesterol + complications 109	vit D							
High Cholesterol + complications 110	Vit D once monthely							
High Cholesterol + complications 111	Vit. D tab.	paracetamol						
High Cholesterol + complications 112	ACE inhibitor							
High Cholesterol + complications 113	ACE inhibitor	Multivitamin tab						
